# The E3/E4 ubiquitin conjugation factor UBE4B interacts with and ubiquitinates the HTLV-1 Tax oncoprotein to promote NF-κB activation

**DOI:** 10.1371/journal.ppat.1008504

**Published:** 2020-12-23

**Authors:** Suchitra Mohanty, Teng Han, Young Bong Choi, Alfonso Lavorgna, Jiawen Zhang, Edward William Harhaj

**Affiliations:** 1 Department of Microbiology and Immunology, Penn State College School of Medicine, Hershey, Pennsylvania, United States of America; 2 W. Harry Feinstone Department of Molecular Microbiology and Immunology, Johns Hopkins Bloomberg School of Public Health, Baltimore, Maryland, United States of America; 3 Department of Oncology, Sidney Kimmel Comprehensive Cancer Center, Johns Hopkins School of Medicine, Baltimore, Maryland, United States of America; Imperial College London, UNITED KINGDOM

## Abstract

Human T-cell leukemia virus type 1 (HTLV-1) is the etiological agent of adult T-cell leukemia/lymphoma (ATLL), and the neurological disease HTLV-1-associated myelopathy/tropical spastic paraparesis (HAM/TSP). The HTLV-1 Tax protein persistently activates the NF-κB pathway to enhance the proliferation and survival of HTLV-1 infected T cells. Lysine 63 (K63)-linked polyubiquitination of Tax provides an important regulatory mechanism that promotes Tax-mediated interaction with the IKK complex and activation of NF-κB; however, the host proteins regulating Tax ubiquitination are largely unknown. To identify new Tax interacting proteins that may regulate its ubiquitination we conducted a yeast two-hybrid screen using Tax as bait. This screen yielded the E3/E4 ubiquitin conjugation factor UBE4B as a novel binding partner for Tax. Here, we confirmed the interaction between Tax and UBE4B in mammalian cells by co-immunoprecipitation assays and demonstrated colocalization by proximity ligation assay and confocal microscopy. Overexpression of UBE4B specifically enhanced Tax-induced NF-κB activation, whereas knockdown of UBE4B impaired Tax-induced NF-κB activation and the induction of NF-κB target genes in T cells and ATLL cell lines. Furthermore, depletion of UBE4B with shRNA resulted in apoptotic cell death and diminished the proliferation of ATLL cell lines. Finally, overexpression of UBE4B enhanced Tax polyubiquitination, and knockdown or CRISPR/Cas9-mediated knockout of UBE4B attenuated both K48- and K63-linked polyubiquitination of Tax. Collectively, these results implicate UBE4B in HTLV-1 Tax polyubiquitination and downstream NF-κB activation.

## Author summary

Infection with the retrovirus HTLV-1 leads to the development of either CD4+CD25+ leukemia/lymphoma (ATLL) or a demyelinating neuroinflammatory disease (HAM/TSP) in a subset of infected individuals. The HTLV-1 Tax protein is a regulatory protein which regulates viral gene expression and persistently activates cellular signaling pathways such as NF-κB to drive the clonal expansion and longevity of HTLV-1 infected CD4+ T cells. Polyubiquitination of Tax is a key mechanism of NF-κB activation by assembling and activating IκB kinase (IKK) signaling complexes; however, the host factors regulating Tax ubiquitination have remained elusive. Here, we have identified the E3/E4 ubiquitin conjugation factor UBE4B as a novel Tax binding protein that promotes both K48- and K63-linked polyubiquitination of Tax. Knockdown or knockout of UBE4B impairs Tax-induced NF-κB activation and triggers apoptosis of HTLV-1-transformed cells. Therefore, UBE4B is an integral host factor that supports HTLV-1 Tax polyubiquitination, NF-κB activation and cell survival.

## Introduction

Human T-cell leukemia virus type 1 (HTLV-1) is an oncogenic retrovirus estimated to infect between 5 and 10 million people worldwide [1]. Highly endemic areas of HTLV-1 include southern Japan, sub-Saharan Africa, South America, the Caribbean and the Middle East. Recent epidemiological studies have found >40% of adults in indigenous communities in Central Australia infected with HTLV-1 [2]. HTLV-1 predominantly infects CD4+ T cells and infection is associated with the development of a CD4+CD25+ malignancy, adult T-cell leukemia/lymphoma (ATLL), in ~5% of infected individuals after a long latent period (about 60 years) [3]. HTLV-1 infection can also lead to inflammatory diseases such as HTLV-1-associated myelopathy/tropical spastic paraparesis (HAM/TSP) [4].

The HTLV-1 regulatory proteins Tax and HBZ are strongly linked to viral persistence and pathogenesis [5]. Both Tax and HBZ have been ascribed oncogenic functions and together contribute to the survival of HTLV-1 infected cells, and play distinct roles in the genesis and/or maintenance of ATLL. Tax is required for viral gene expression by recruiting CREB/ATF transcription factors and coactivators CBP/p300 to the HTLV-1 5’ long terminal repeat (LTR) [6]. In addition, Tax dysregulates the cell cycle, inhibits tumor suppressors Rb, p53 and DLG1, and activates multiple signaling pathways such as NF-κB to enforce a transcriptional program supporting the proliferation and survival of T cells carrying the HTLV-1 provirus [7,8]. Tax is highly immunogenic and represents a main target of CD8+ T cells, therefore Tax expression and HTLV-1 plus-strand transcription is commonly silenced as a mechanism of immune evasion [9]. However, the HTLV-1 provirus can be reactivated from latency in response to stress stimuli such as hypoxia, p38-MAPK signaling or oxidative stress [10–12]. In ATLL, Tax expression is undetectable in the peripheral blood of ~60% of ATLL cases due to genetic or epigenetic mechanisms [13]; however, HBZ expression remains ubiquitously expressed.

Tax is a potent activator of canonical and noncanonical NF-κB pathways, and persistent activation of NF-κB is a hallmark of ATLL [14]. Tax activates the canonical NF-κB pathway by interacting with the IκB kinase (IKK) complex through direct binding with NEMO/IKKγ to promote the phosphorylation and degradation of the inhibitor IκBα [15]. Tax also persistently activates the noncanonical NF-κB pathway via NEMO binding to promote the processing of the precursor p100/NF-κB2 to the p52 subunit [16]. Modification of Tax by polyubiquitination is tightly linked to NF-κB activation [17,18]. Tax is conjugated with lysine 63 (K63)-linked polyubiquitin (polyUb) chains, resulting in the recruitment of the IKK complex to foci in the vicinity of the cis-Golgi, which serves as a hub for Tax/IKK interaction and IKK activation [19,20]. Tax K63-linked polyubiquitination on multiple carboxyl-terminal lysines facilitates interaction with NEMO and the IKK complex for the subsequent activation of NF-κB [21]. The E2 enzyme Ubc13 plays a critical role in the K63-linked polyubiquitination of Tax and NF-κB activation [22]; however, other host factors directly modulating Tax ubiquitination are largely unknown. The E3 ligase PDLIM2 has been shown to conjugate K48-linked polyUb chains on Tax, however this leads to the proteasomal degradation of Tax in the nuclear matrix [23]. Similarly, the E3 ligase Ring Finger Protein 4 (RNF4) can ubiquitinate and degrade Tax upon treatment of HTLV-1-transformed T cell lines with a combination of arsenic and interferon-α [24]. Tax also interacts with selective autophagy receptors TAX1BP1, Optineurin and SQSTM-1/p62, to potentiate Tax ubiquitination and/or NF-κB activation [25–27], although the role of autophagy in Tax-IKK activation remains poorly understood. Finally, Tax can interact with membrane associated CADM1/TSLC1 to promote K63-linked polyubiquitination of Tax and the inhibition of the ubiquitin-editing enzyme A20 [28].

Tax has also been shown to target E3 ligases for the generation of unanchored polyUb chains or ubiquitination of substrates other than Tax to activate IKK signaling or promote cell survival. Tax activates Ring Finger Protein 8 (RNF8) to induce the assembly of long unanchored K63-linked polyUb chains that activate IKK and TAK1 kinases [29]. Tax has also been shown to recruit the linear (Met1-linked) ubiquitin chain assembly complex (LUBAC) to IKK for the generation of K63/M1-linked hybrid polyUb chains [30]. These hybrid chains may trigger the oligomerization and activation of the IKK complex. Tax interacts with the E3 ligase TRAF6 and promotes its E3 activity, leading to MCL-1 K63-linked polyubiquitination and stabilization and inhibition of apoptosis [31]. Although Tax binds to TRAF6 it is unclear if TRAF6 can conjugate Tax with K63-linked polyUb chains. Taken together, ubiquitination plays substantial roles in Tax-induced IKK activation and oncogenesis.

UBE4B is a human homolog of the *Saccharomyces cerevisiae* UFD2. Yeast UFD2, encoded by a single-copy gene, is the first reported E4 ubiquitination factor and is required for elongation of an oligoubiquitin chain on certain substrates that are subsequently recognized by the 26S proteasome for degradation [32,33]. UFD2 is involved in the endoplasmic reticulum-associated degradation (ERAD) pathway by interacting with the AAA-type ATPase Cdc48 [34]. UBE4B and its homologs share a 70-amino acid U-box domain that confers ligase activity. Sequence profile alignments indicate that the U-box is a derived version of the RING finger domain, but the signature cysteines in the RING finger domain that are responsible for metal chelating, are not conserved in the U-box [35]. However, the predicted structure of the U-box is very similar to that of the RING finger domain [36], which indicates that U-box proteins may also have the capability to function as E3 ligases independently. Indeed, UFD2 can function as a bona fide E3 ubiquitin ligase to promote ubiquitin conjugation of unfolded proteins [37]. Thus, UBE4B and its homologs can clearly function as E3 ligases, and their E4 function may represent a specialized type of E3 activity with mono- or oligoubiquitinated proteins as substrates. UBE4B has been identified as an E3/E4 ligase that collaborates with Hdm2 to catalyze the polyubiquitination and degradation of the tumor suppressor p53 [38]. UBE4B has also been shown to ubiquitinate additional substrates such as EGFR, Ataxin-3 and OTUB1 [39–41]. To date, UBE4B has not been implicated in the ubiquitination of any viral proteins.

In this study, we undertook an approach to identify new Tax binding proteins that may regulate Tax polyubiquitination and NF-κB activation. We have identified the E3/E4 ubiquitin conjugation factor UBE4B as a novel Tax binding protein which promotes both K63- and K48-linked Tax polyubiquitination and NF-κB activation.

## Results

### Tax interacts with UBE4B

**Fig 1:**
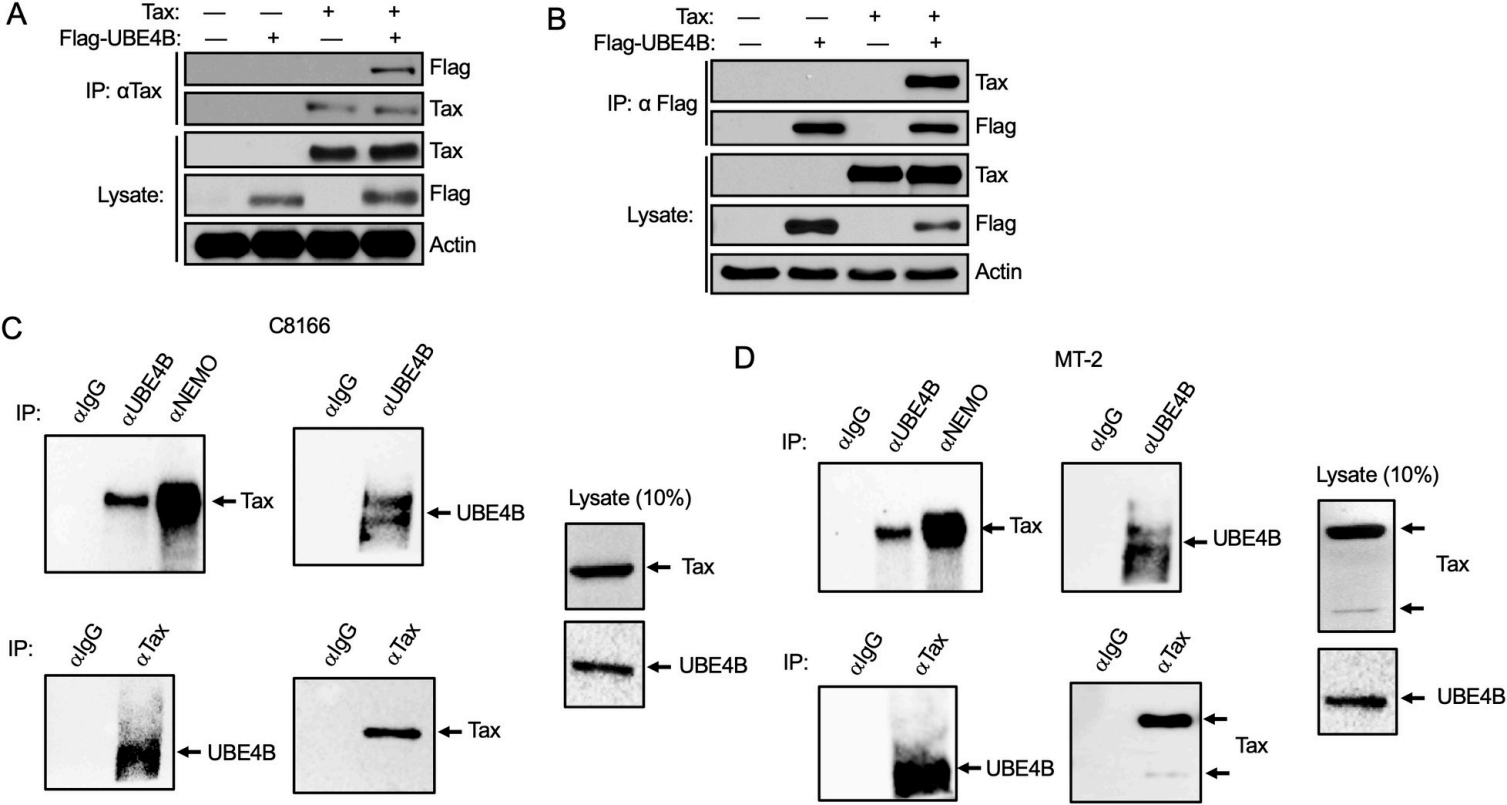
Tax interacts with UBE4B. (A-B) Co-IP analysis with either Tax (A) or Flag-UBE4B (B) immunoprecipitates from lysates of 293T cells transfected with the indicated plasmids. (C-D) Co-IP analysis with either control IgG, anti-UBE4B, anti-NEMO or anti-Tax immunoprecipitates from lysates of C8166 (C) and MT-2 (D) cells as indicated.

In order to identify new binding partners of Tax, we conducted a yeast two-hybrid screen using full-length Tax as bait with a cDNA library derived from human leukocytes and activated mononuclear cells. Importantly, known Tax interacting proteins DLG1 [42], NF-κB2 [16] and TAX1BP3 [43] were identified in the screen. A potential novel Tax binding protein that emerged from this screen was the E3/E4 ubiquitin conjugation factor UBE4B. A UBE4B fragment spanning amino acids 70–347 was identified in the screen. To confirm the interaction between Tax and UBE4B, a co-immunoprecipitation (co-IP) experiment was performed with lysates from 293T cells transfected with Tax and a Flag-tagged UBE4B plasmid. As shown in Fig 1A, UBE4B interacted with Tax when both proteins were overexpressed. The reciprocal IP in which UBE4B was immunoprecipitated also confirmed Tax and UBE4B interaction (Fig 1B). The interaction was further examined by co-IPs using Tax and a UBE4B catalytically inactive mutant, in which the highly conserved proline at position 1140 was replaced with alanine (UBE4B P1140A) [44]. The dominant-negative mutant (UBE4B P1140A) was still able to interact with Tax (S1A Fig). Tax point mutants M22 and M47 are impaired in NF-κB and CREB activation, respectively [22]. UBE4B interacted with Tax M22 and M47 mutants, similar to Tax WT (S1B Fig).

We next performed co-IPs using HTLV-1-transformed cell lines C8166 and MT-2 to determine if endogenous Tax and UBE4B proteins could interact. The anti-UBE4B-immunoprecipitated complex was immunoblotted with anti-Tax. As a positive control, NEMO was immunoprecipitated and immunoblotted with anti-Tax. As expected, NEMO and Tax strongly interacted in both C8166 and MT-2 cells (Fig 1C and 1D). UBE4B was also found to interact with Tax using lysates from C8166 and MT-2 cells (Fig 1C and 1D). A reciprocal IP in which Tax was immunoprecipitated also confirmed the interaction between Tax and UBE4B in C8166 and MT-2 cells (Fig 1C and 1D). Therefore, UBE4B interacts with Tax under both overexpression and endogenous conditions.

### UBE4B and Tax colocalize in HTLV-1-transformed cells

**Fig 2:**
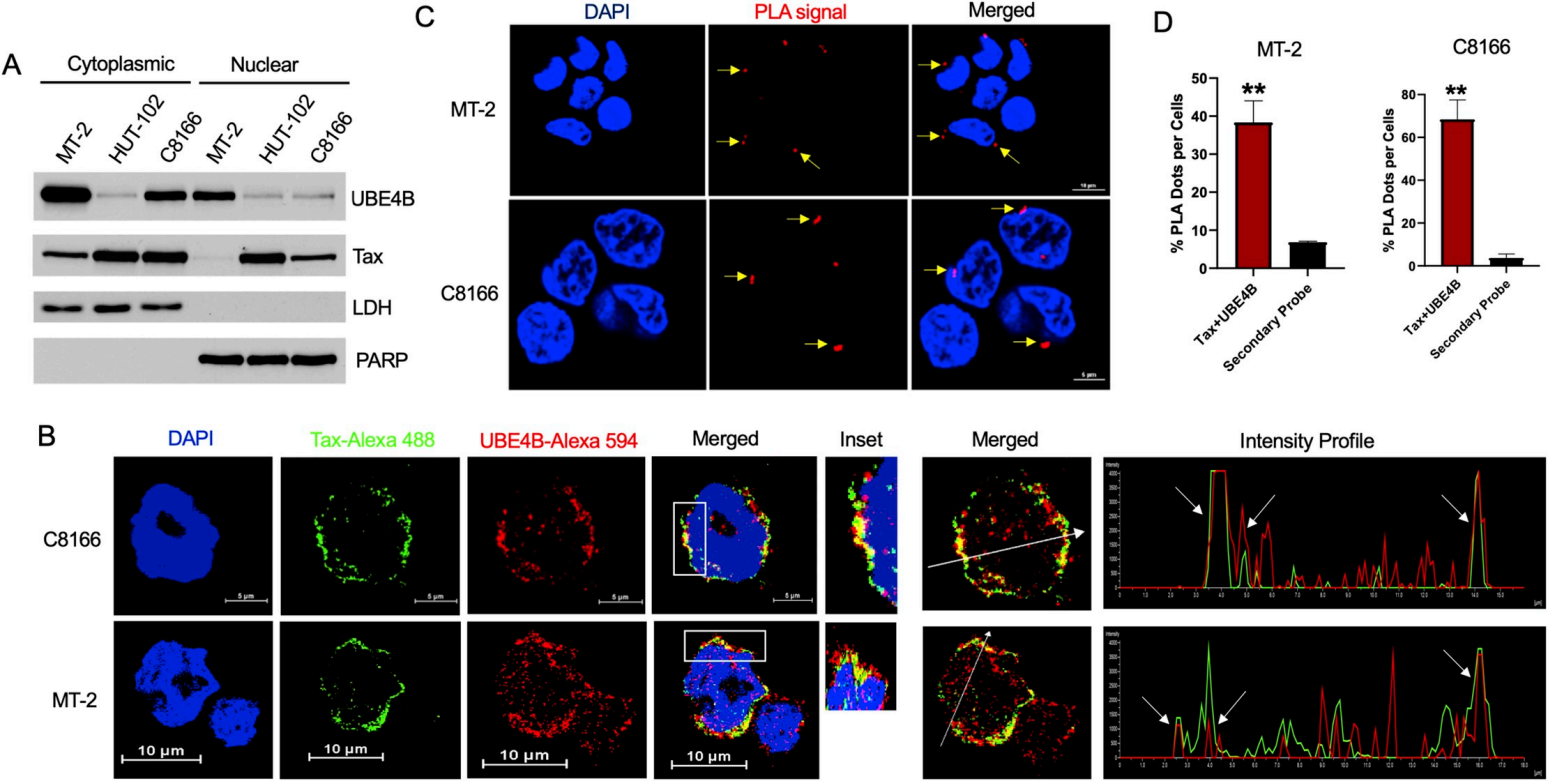
Co-localization of UBE4B and Tax in HTLV-1-transformed cells. (A) Immunoblotting was performed with the indicated antibodies using cytoplasmic and nuclear fractions obtained from MT-2, HUT-102 and C8166 cells. (B) Immunofluorescence (IF) confocal microscopy was performed using MT-2 and C8166 cells with the indicated antibodies. The fluorescence intensity profile was plotted along the white arrow crossing the nucleus and represented in the graph showing overlap using NIS Element software. (C) PLA was performed using MT-2 and C8166 cells with Tax and UBE4B antibodies. (D) Graphical representation of the number of PLA signals with the secondary probe stained sample as a negative control. Unpaired Student’s *t*-test, **P <0.01.

Tax shuttles between the cytoplasm and nucleus and is distributed in both compartments at steady state [45]. Similarly, UBE4B localizes in both the cytoplasm and nucleus [39], therefore we sought to determine where Tax and UBE4B interacted in cells. First, we fractionated MT-2, HUT-102 and C8166 cells into cytoplasmic and nuclear fractions, and examined UBE4B and Tax expression. Lactate dehydrogenase (LDH) and poly (ADP-ribose) polymerase (PARP) immunoblotting confirmed the purity of cytoplasmic and nuclear fractions, respectively. As expected, Tax was expressed in both cytoplasmic and nuclear compartments in the cell lines (Fig 2A). UBE4B was found predominantly in cytoplasmic fractions, but was also detected in nuclear fractions (Fig 2A). Next, MT-2 and C8166 cells were subjected to immunostaining and confocal microscopy. Tax strongly co-localized with UBE4B, predominantly in a perinuclear region, in both MT-2 and C8166 cells (Fig 2B). We also plotted colocalization intensity profiles and generated 3D projections of z-stack images (S1 and S2 Movies), which further supported Tax-UBE4B colocalization. As a negative control, Jurkat cells were subjected to immunostaining with the same antibodies, and as expected there was no staining with anti-Tax (S2A Fig). To further confirm the Tax-UBE4B interaction, we conducted an in situ proximity ligation assay (PLA), which can detect proteins found within 40 nm of each other. PLA signals were only observed in MT-2 and C8166 cells when both Tax and UBE4B antibodies were used together with secondary probes (Fig 2C). PLA signals were found mostly in the cytoplasm, and in a perinuclear region. PLA signals were quantified, confirming a significant increase compared to secondary probe alone (Fig 2D). As a negative control, the PLA assay was performed in Jurkat cells with identical antibodies, and as expected no PLA signals were detected (S2B Fig). Collectively, these data provide strong evidence of an interaction between endogenous Tax and UBE4B.

### UBE4B enhances Tax-mediated activation of NF-κB

**Fig 3:**
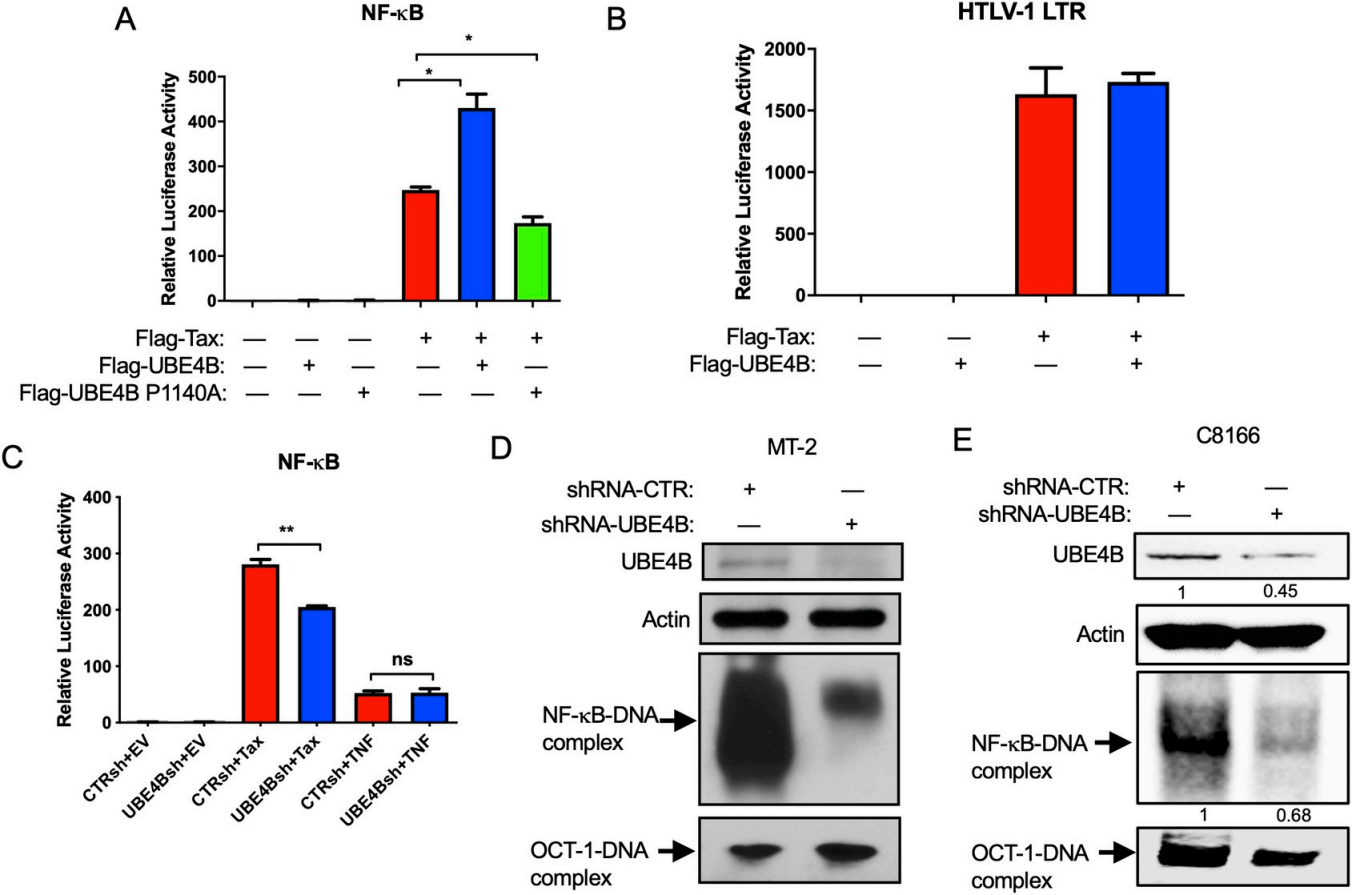
UBE4B enhances Tax-mediated activation of NF-κB. (A-B) NF-κB and HTLV-1 LTR luciferase assays in 293T cells transfected with NF-κB-TATA or HTLV-1 luciferase, pRL-tk, Flag-UBE4B, FLAG-UBE4B P1140A and Flag-Tax as indicated. (C) NF-κB luciferase assays in 293T cells expressing either control shRNA or UBE4B shRNA were transfected with NF-κB-TATA luciferase, pRL-tk, Flag-Tax or stimulated with TNF for 8 h as indicated. (D-E) NF-κB and OCT-1 EMSA using nuclear extracts from MT-2 (D) and C8166 (E) cells expressing either control shRNA or UBE4B shRNA. C8166 cells were treated with Dox to induce UBE4B shRNA. UBE4B knockdown was confirmed by immunoblotting. Unpaired Student’s *t*-test, *P <0.05, **P <0.01, ns = not significant.

Since UBE4B and Tax mainly interacted in the cytoplasm, where Tax activates IKK, we hypothesized that UBE4B may regulate Tax-induced NF-κB activation. To test this hypothesis, 293T cells were transfected with Flag-Tax together with Flag-UBE4B or catalytically inactive Flag-UBE4B P1140A, and NF-κB luciferase assays were performed. As expected, Tax expression resulted in potent activation of the NF-κB luciferase reporter (Fig 3A). Transfection of wild-type UBE4B, but not catalytically inactive UBE4B P1140A, significantly enhanced Tax-mediated NF-κB activation (Fig 3A). Wild-type UBE4B or UBE4B P1140A alone did not activate NF-κB (Fig 3A). In contrast, overexpression of UBE4B had no effect on Tax-mediated HTLV-1 LTR activation, therefore UBE4B appears to selectively modulate Tax activation of NF-κB (Fig 3B).

To further examine the functional roles of the UBE4B-Tax interaction, UBE4B expression was suppressed with three independent shRNAs in 293T cells using recombinant shRNA expressing lentiviruses (S3A Fig). UBE4B shRNA3 was most effective at reducing UBE4B protein expression (S3A Fig), and was used for subsequent experiments. Tax-induced NF-κB activation was significantly decreased in 293T cells expressing UBE4B shRNA (Fig 3C), and UBE4B potentiation of NF-κB appeared to be specific for Tax since UBE4B depletion had no effect on tumor necrosis factor (TNF)-induced NF-κB activation (Fig 3C).

We next sought to determine if UBE4B supported persistent NF-κB activation by Tax in HTLV-1-transformed cells. To this end, we knocked down UBE4B using lentiviral-expressed shRNA described above, and also generated stable cell lines expressing doxycycline (Dox)-inducible UBE4B shRNAs expressed by the SMARTvector inducible lentiviral shRNA vector (Horizon). This vector also expresses TurboGFP upon treatment with Dox to facilitate the identification of cells inducibly expressing shRNAs. Three different shRNAs were screened for endogenous UBE4B knockdown in 293T cells after induction with Dox. Both shRNAs #2 and #3 effectively suppressed endogenous UBE4B protein expression (S3B Fig), however the vector expressing shRNA #2 exhibited greater GFP inducibility as shown by Incucyte S3 live-cell analysis (S3C Fig). Therefore, control scrambled and shRNA #2 lentiviral vectors were used to generate C8166, MT-2 and TL-OM1 (Tax-) cells stably expressing inducible control and UBE4B shRNAs. These cell lines were then treated with Dox and GFP expression analyzed by fluorescence microscopy, revealing GFP expression in the majority of cells (S3D Fig).

To examine a potential role of UBE4B in NF-κB activation in ATLL cell lines, UBE4B was knocked down with shRNA in MT-2 and C8166 cells and nuclear extracts were subjected to an NF-κB electrophoretic mobility shift assay (EMSA). Immunoblotting with lysates from MT-2 and C8166 cells expressing UBE4B shRNAs confirmed UBE4B knockdown (Fig 3D and 3E). As expected, robust NF-κB DNA binding was observed with MT-2 and C8166 cells expressing control shRNA; however, shRNA-mediated knockdown of UBE4B in MT-2 and C8166 cells significantly impaired NF-κB but not OCT-1 DNA binding (Fig 3D and 3E).

**Fig 4:**
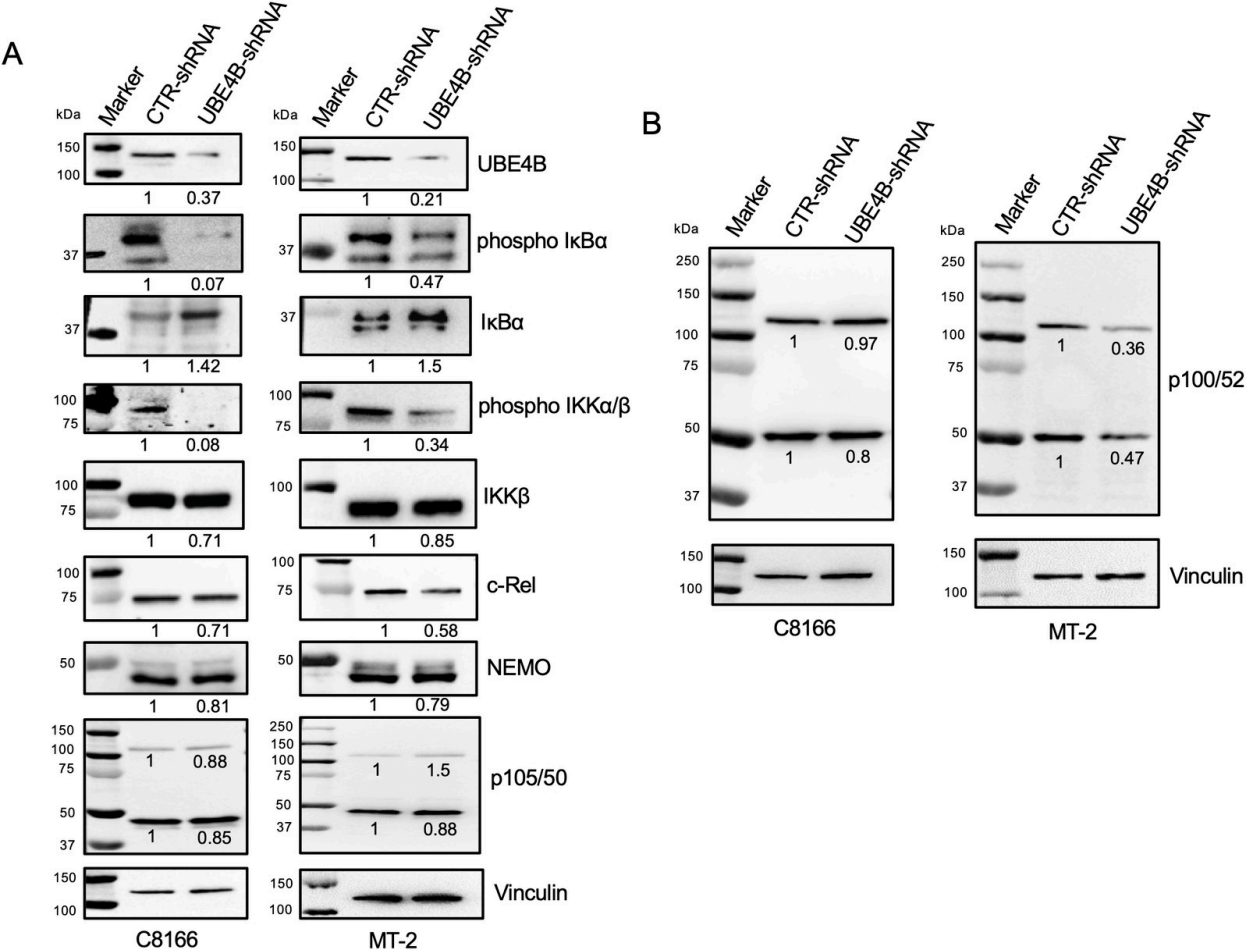
UBE4B regulates IKK and canonical NF-κB signaling in HTLV-1-transformed cells. (A-B) UBE4B was inducibly knocked down in C8166 and MT-2 cells by Dox treatment and immunoblotting was performed using lysates with the indicated antibodies. Canonical NF-κB and IKK activation was examined in panel A and noncanonical NF-κB activation was examined in panel B.

To further understand how UBE4B regulated NF-κB signaling in HTLV-1-transformed cells, we knocked down UBE4B by Dox treatment in C8166 and MT-2 cells stably expressing inducible UBE4B shRNA. Knockdown of UBE4B was confirmed by immunoblotting (Fig 4A). We next examined the activation of IKK and the expression of NF-κB subunits by immunoblotting. Knockdown of UBE4B in both C8166 and MT-2 cells suppressed the activation of IKK and phosphorylation of IκBα, as determined by phospho-specific antibodies (Fig 4A). Consistent with these findings, total IκBα expression was increased due to its impaired phosphorylation and degradation (Fig 4A). Therefore, UBE4B functions at the level of or upstream of the IKK complex. The expression of the NF-κB subunit c-Rel was modestly decreased upon UBE4B knockdown (Fig 4A), likely due to impaired induction by Tax and RelA/p65 [46]. However, expression of IKKβ, NEMO and p105/p50 were comparable after UBE4B knockdown (Fig 4A). We next examined noncanonical NF-κB activation in C8166 and MT-2 cells upon UBE4B knockdown. However, the processing of p100 to p52 was not impacted by UBE4B knockdown (Fig 4B), indicating that UBE4B specifically supports canonical NF-κB activation by Tax. Given the importance of UBE4B in IKK activation in HTLV-1-transformed cells, we next examined if UBE4B could interact with NEMO by co-IP experiments. However, co-IPs did not detect a potential interaction between UBE4B and NEMO (S4 Fig). Nevertheless, these experiments do not exclude the possibility of a potentially weak or transient interaction between UBE4B and NEMO in these cells.

### Knockdown of UBE4B impairs Tax-induced expression of NF-κB target genes

**Fig 5:**
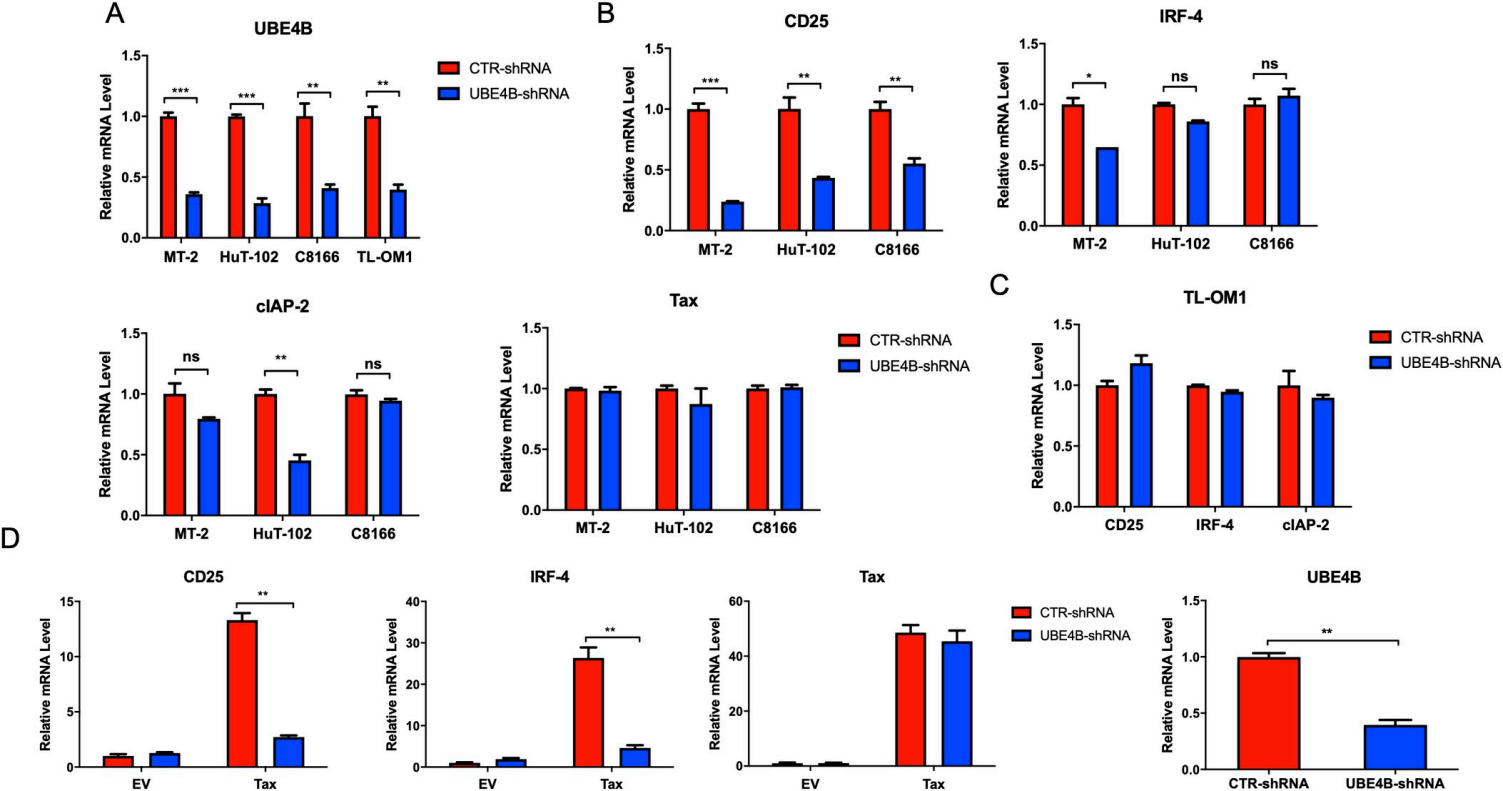
Knockdown of UBE4B impairs Tax-induced NF-κB target gene expression. (A-C) qRT-PCR of UBE4B, CD25, IRF-4, cIAP-2 and Tax mRNAs in MT-2, HUT-102, C8166 and TL-OM1 cells expressing control shRNA or UBE4B shRNA. (D) qRT-PCR of CD25, IRF-4, Tax and UBE4B mRNAs in Jurkat T cells expressing control shRNA or UBE4B shRNA and transduced with lentivirus expressing Tax. Unpaired Student’s *t*-test, *P <0.05, **P <0.01, ***P <0.001, ns = not significant.

The HTLV-1-transformed cell lines MT-2, HUT-102 and C8166 have high levels of Tax expression and persistent NF-κB activation. Given that Tax expression is silenced in ~60% of ATLL [13], we wondered if UBE4B supported NF-κB activation in Tax-negative ATLL cells. The ATLL cell line TL-OM1 is derived from an ATLL patient and lacks Tax expression. Although Tax is not expressed in these cells, persistent NF-κB activation is still maintained by genetic and/or epigenetic changes [47]. To confirm the effects of UBE4B on Tax-mediated NF-κB activation, the mRNA expression of NF-κB target genes was analyzed in Tax+ and Tax- ATLL cell lines by qRT-PCR after shRNA-mediated knockdown of UBE4B. UBE4B knockdown was confirmed by qRT-PCR in Tax+ MT-2, HUT-102, C8166 and Tax- TL-OM1 cell lines (Fig 5A). CD25 is the high affinity subunit of the IL-2 receptor and is critical for T-cell proliferation. UBE4B knockdown significantly impaired the expression of CD25 in Tax+ MT-2, HUT-102 and C8166 cell lines, but not in Tax- TL-OM1 cells (Fig 5B and 5C). Expression of IRF-4, a transcription factor critical for the survival of ATLL cell lines [48], was significantly decreased in MT-2, but not in HUT-102, C8166 or TL-OM1 cells upon UBE4B depletion (Fig 5B and 5C). Expression of cIAP-2, an anti-apoptotic NF-κB target gene, was significantly decreased in HUT-102, but not in MT-2, C8166 or TL-OM1 cells when UBE4B was knocked down (Fig 5B). It is unclear why UBE4B knockdown did not impact IRF-4 or cIAP-2 expression in all the Tax+ cell lines, but may reflect cell-type differences in the regulation of these genes. Importantly, Tax expression remained constant in MT-2, HUT-102 or C8166 cells upon UBE4B knockdown (Fig 5B).

To further confirm the effect of UBE4B on Tax-mediated NF-κB activation, UBE4B was knocked down in Jurkat T cells and Tax was introduced into the cells by lentiviral transduction. Cells were lysed two days later and mRNA was extracted for qRT-PCR experiments. The expression of both CD25 and IRF-4 were significantly reduced after UBE4B knockdown in Jurkat T cells expressing Tax (Fig 5D). However, Tax expression was comparable between the UBE4B knockdown and control samples (Fig 5D). UBE4B mRNA was significantly downregulated by the shRNA (Fig 5D). Taken together, UBE4B specifically mediates Tax-induced NF-κB activation and does not play a role in NF-κB activation in Tax- ATLL cells.

### UBE4B expression is not regulated by Tax or NF-κB

We considered the possibility that Tax may upregulate UBE4B expression to potentiate NF-κB signaling. To test this hypothesis, Jurkat Tax Tet-on T cells were treated with Dox to induce Tax expression and after two days mRNA was extracted for qRT-PCR analysis. As expected, Tax upregulated the expression of CD25, however UBE4B expression was not increased by Tax (S5A Fig). Next, UBE4B expression was examined in a panel of HTLV-1-transformed cell lines that all exhibit persistent NF-κB activation. UBE4B mRNA (S5B Fig) and protein (S5C Fig) displayed variation in the panel of Tax+ (MT-2, HUT-102, C8166, MT-4) and Tax- (TL-OM1, ED-40515(-) and ATL-43T) ATLL cell lines, as well as control Jurkat T cells and peripheral blood mononuclear cells (PBMCs) from a normal donor. However, the differences did not correlate with Tax expression (S5C Fig). Therefore, UBE4B expression does not appear to be regulated by Tax or NF-κB.

### CRISPR/Cas9-mediated knockout of UBE4B impairs Tax-induced NF-κB activation

To further corroborate the UBE4B shRNA results on Tax-NF-κB activation, we next used CRISPR/Cas9 to generate UBE4B knockout (KO) 293T cells. Three different gRNAs targeting *Ube4b* exon 10 were expressed in pLentiCRISPRv2 for the production of recombinant lentiviruses expressing UBE4B gRNAs and Cas9. 293T cells were transduced with lentiviruses and the bulk population analyzed by immunoblotting for UBE4B knockout. Only one of the three gRNAs (gRNA3) resulted in efficient UBE4B knockout, and therefore cells expressing either UBE4B gRNA3 or control gRNA were subjected to limiting dilution to isolate individual clones. Three individual KO clones (G12, H1 and F5) and two control clones (E2 and H10) were examined for UBE4B expression by immunoblotting and genomic DNA sequencing of *Ube4b* exon 10. UBE4B knockout clones G12 and H1 both harbored an insertion of an adenine in *Ube4b* exon 10 which would result in a frameshift mutation (S6A Fig). All three KO clones lacked expression of UBE4B as determined by immunoblotting (S6B Fig).

**Fig 6:**
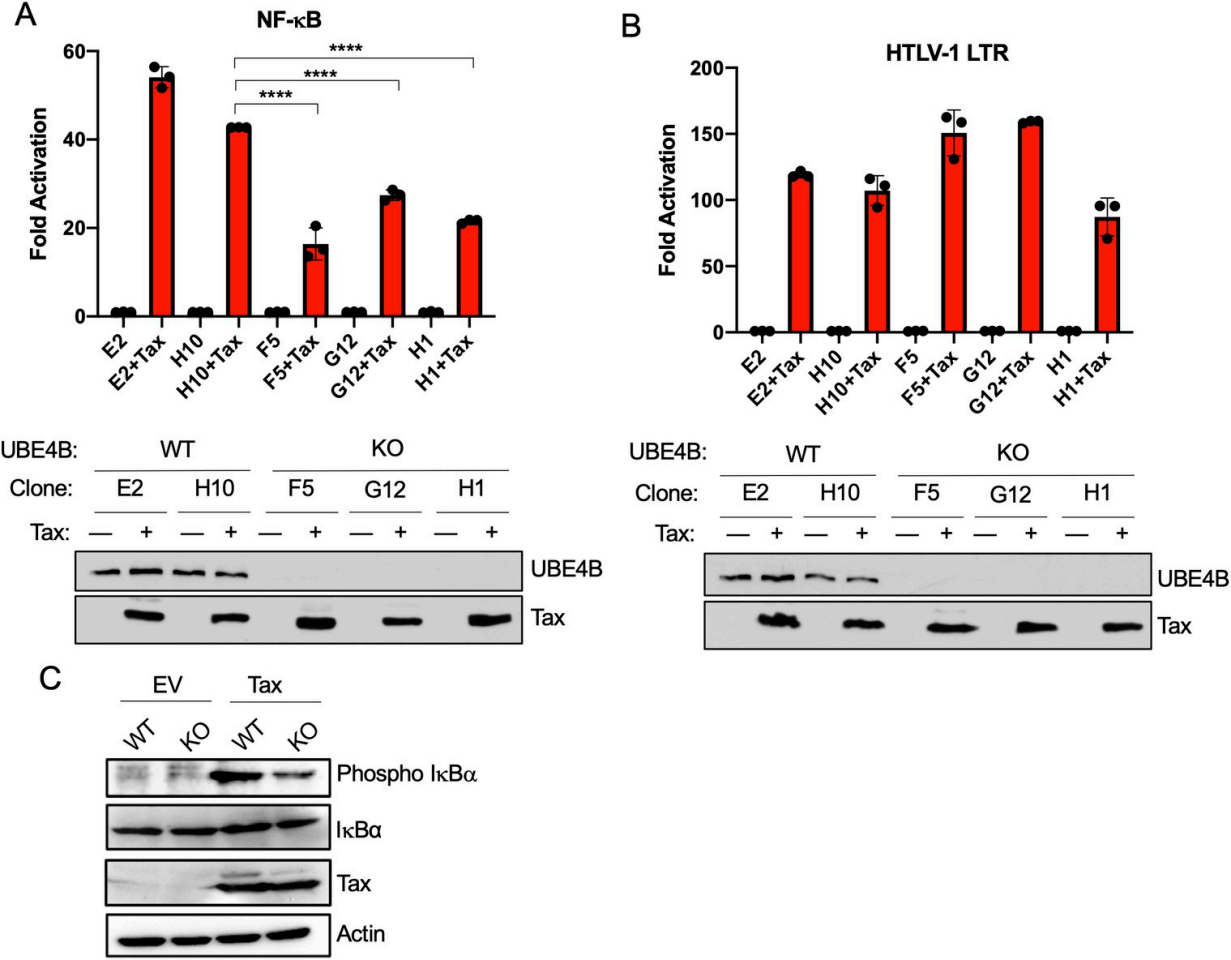
Tax activation of NF-κB is impaired in UBE4B KO cells. (A-B) NF-κB and HTLV-1 LTR luciferase assays in wild-type (E2, H10) and UBE4B KO (F5, G12, H1) 293T cells transfected with NF-κB-TATA or HTLV-1 LTR luciferase, pRL-tk, and Tax as indicated. Immunoblotting was performed with lysates from transfected cells. (C) Immunoblotting was performed with the indicated antibodies using lysates from wild-type and UBE4B KO (clone H1) 293T cells transfected with Tax. One-way ANOVA with Dunnett’s post hoc test, ****P <0.0001.

To examine Tax-induced NF-κB activation in UBE4B KO 293T cells, control and UBE4B KO clones were transfected with a Tax plasmid and subjected to NF-κB and HTLV-1 LTR luciferase assays. Tax activation of NF-κB was significantly reduced in all three UBE4B KO clones; however, Tax activation of the HTLV-1 LTR was either increased or unchanged in control and UBE4B KO clones (Fig 6A and 6B). Tax expression was comparable in each of the transfected clones (Fig 6A and 6B). We next examined phosphorylation of the NF-κB inhibitor IκBα using a phospho-specific antibody. Phosphorylation of IκBα in response to Tax expression was decreased in UBE4B KO cells further confirming our earlier results that UBE4B functions at the level of or upstream of the IKK complex (Fig 6C).

### Knockdown of UBE4B promotes apoptosis of HTLV-1-transformed cell lines

**Fig 7:**
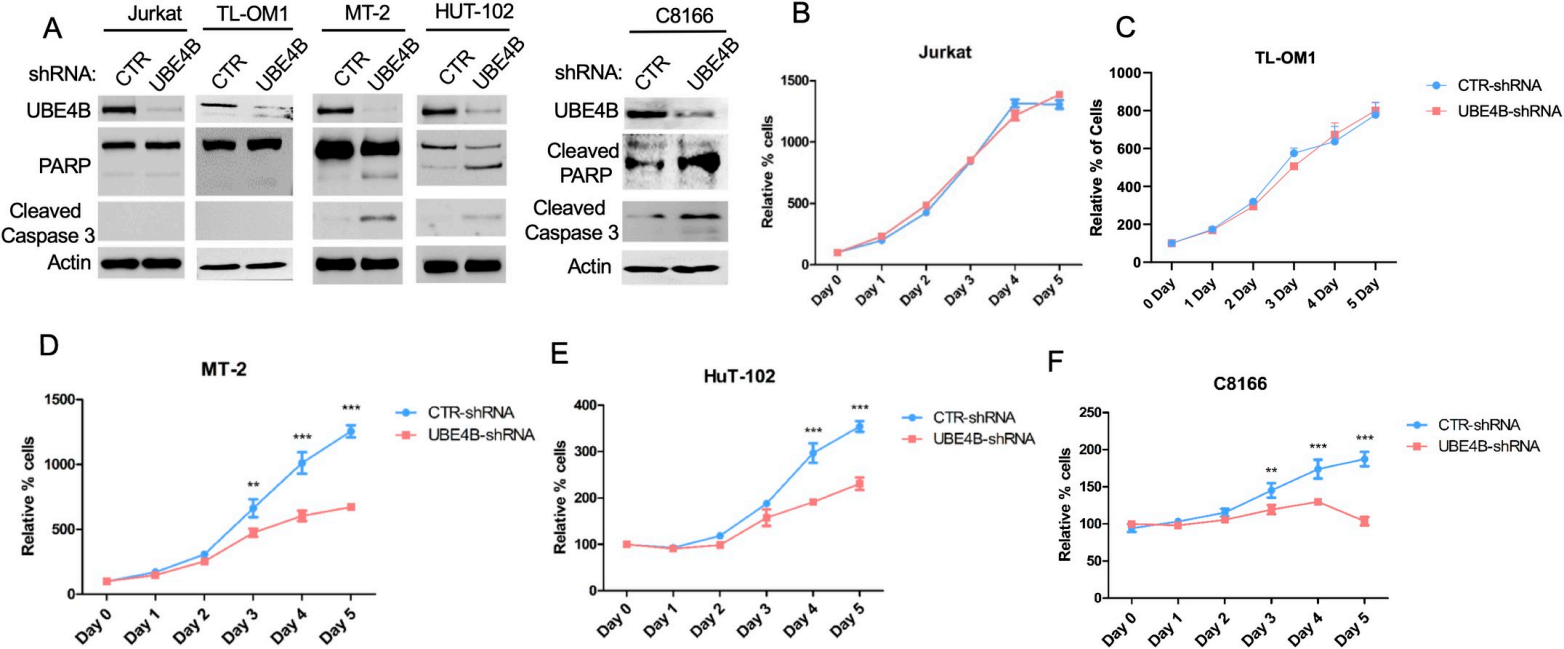
Knockdown of UBE4B promotes apoptotic cell death in HTLV-1-transformed cell lines. (A) Immunoblotting was performed with the indicated antibodies using lysates from Jurkat, TL-OM1, MT-2, HUT-102 and C8166 cells expressing control shRNA or UBE4B shRNA. C8166 and TL-OM1 cells were treated with Dox to induce UBE4B shRNA. (B-F) Cell viability assay was performed at the indicated times with Jurkat, TL-OM1, MT-2, HUT-102 and C8166 cells expressing control shRNA or UBE4B shRNA. TL-OM1 cells were treated with Dox to induce UBE4B shRNA. Unpaired Student’s *t*-test, **P <0.01, ***P <0.001.

Given that NF-κB is essential for HTLV-1-induced oncogenesis and cell survival, we next examined if UBE4B depletion with shRNA would trigger apoptosis of HTLV-1-transformed cells. Indeed, knockdown of UBE4B in MT-2, HUT-102 and C8166 cells, but not in control Jurkat cells or Tax- TL-OM1 cells, yielded cleaved forms of PARP and caspase 3 (Fig 7A), indicative of apoptotic cell death. We next examined the effect of UBE4B knockdown on the viability and proliferation of control Jurkat cells, Tax+ HTLV-1-transformed cell lines MT-2, HUT-102 and C8166, and the Tax- ATLL cell line TL-OM1 using CellTiter-Glo luminescent cell viability assay, which measures metabolically active cells by quantifying ATP levels. UBE4B was knocked down with shRNA in Jurkat, MT-2, HUT-102, C8166 and TL-OM1 cells, and metabolically active cells were quantified every 24 h for 5 consecutive days to measure cell proliferation. As expected, cells expressing control scrambled shRNA proliferated vigorously throughout the time course (Fig 7B–7F). However, the proliferation of MT-2, HUT-102 and C8166 cells, but not Jurkat or TL-OM1, was significantly impaired upon UBE4B knockdown (Fig 7B–7F). Therefore, only Tax+ HTLV-1-transformed cell lines are dependent on UBE4B for proliferation and survival.

### UBE4B does not promote Rb and p53 degradation in HTLV-1-transformed cells

The retinoblastoma (Rb) protein regulates a number of key cellular processes, including cell division, differentiation, senescence and apoptosis. It was reported that Tax can directly associate with and target Rb for proteasomal degradation [49]. UBE4B may potentially be recruited to Rb by Tax and function as an E3/E4 ligase to catalyze K48-linked polyubiquitination and degradation of Rb. To test this notion, UBE4B was knocked down with shRNA in Jurkat, MT-2, C8166 and HUT-102 cells, and lysates were subjected to immunoblotting to examine Rb expression. Consistent with the previous study, low levels of Rb protein were observed in HTLV-1-transformed cell lines MT-2, C8166 and HUT-102, compared to control Jurkat T cells (S7 Fig). However, Rb protein expression was unchanged upon UBE4B knockdown in MT-2, C8166 and HUT-102 cells, indicating that UBE4B does not regulate Tax-induced Rb degradation (S7 Fig).

UBE4B extends polyUb chains initiated by Hdm2 to induce the degradation of p53 [38]. To examine whether UBE4B regulated p53 in HTLV-1-transformed cell lines, p53 protein expression was monitored by immunoblotting after UBE4B knockdown. However, the expression of p53 protein was unchanged in MT-2, C8166 and HUT-102 cells after UBE4B knockdown (S7 Fig). Therefore, UBE4B does not appear to regulate p53 stability in HTLV-1-transformed cell lines, suggesting that p53 ubiquitination by UBE4B may be cell-type specific or Tax uses distinct mechanisms to inhibit p53.

### UBE4B is required for Tax polyubiquitination

**Fig 8:**
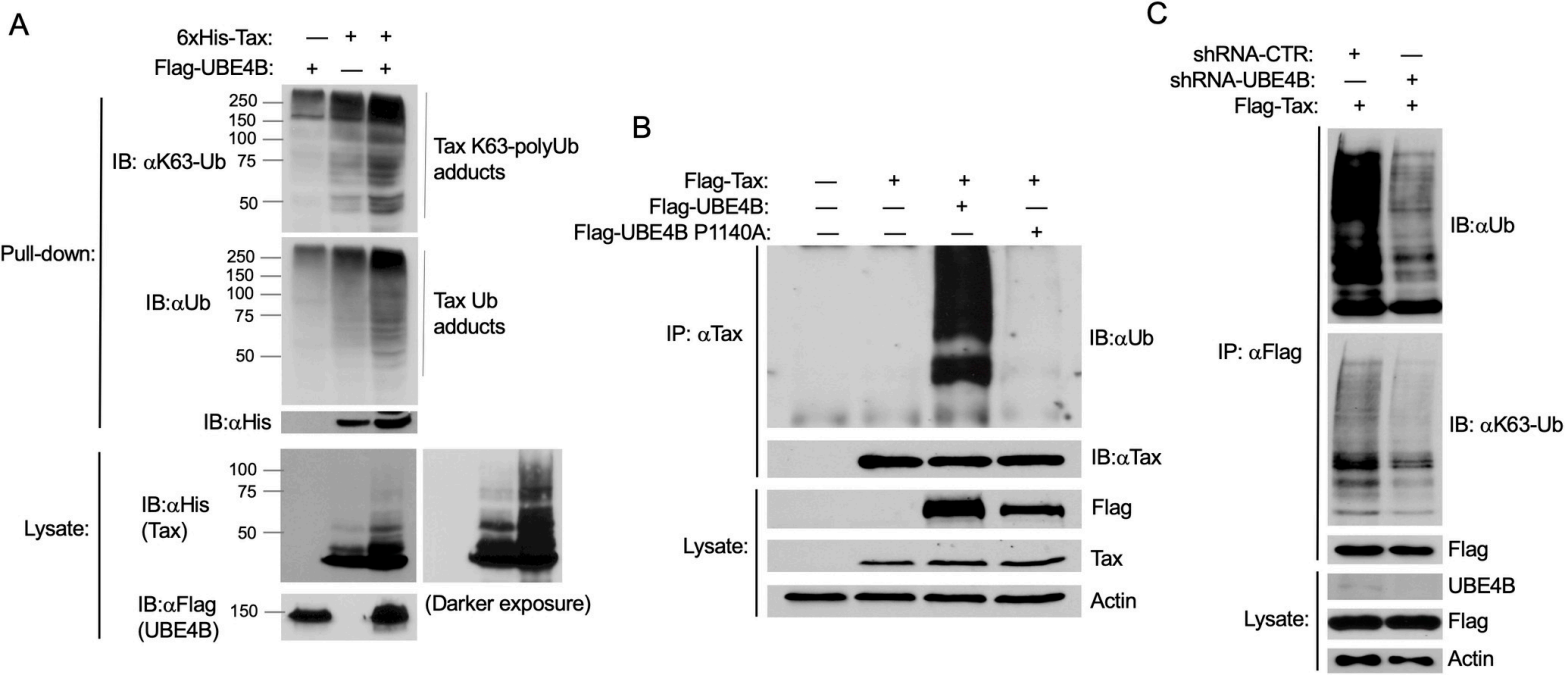
UBE4B promotes the polyubiquitination of Tax. (A) Ubiquitination assay was performed with purified 6xHis-Tax from lysates of 293T cells transfected with 6xHis-Tax and Flag-UBE4B as indicated. Immunoblotting was performed with the indicated antibodies. (B) Ubiquitination assay was performed with Tax immunoprecipitates from lysates of 293T cells transfected with Flag-Tax, Flag-UBE4B and Flag-UBE4B P1140A. (C) Ubiquitination assay was performed with Flag-Tax immunoprecipitates from lysates of 293T cells expressing control shRNA or UBE4B shRNA and transfected with Flag-Tax.

Since UBE4B is an E3/E4 ubiquitin ligase and it potentiates Tax-mediated NF-κB activation, we next examined whether overexpression of UBE4B promotes Tax ubiquitination. 6xHis-Tax was transfected into cells, with and without Flag-UBE4B, and lysates were used to purify Tax with Ni-NTA agarose. Tax polyUb chains were assessed by immunoblotting for total ubiquitin (Ub) and K63-linked Ub. Overexpressed UBE4B enhanced Tax total polyUb and K63-linked polyUb chains (Fig 8A). The catalytic activity of UBE4B was required since the UBE4B P1140A mutant was impaired in enhancing Tax polyUb chains (Fig 8B). We next performed loss of function studies using 293T cells expressing Flag-Tax, and either control shRNA or UBE4B shRNA. Both total and K63-linked polyubiquitination of Tax were impaired when UBE4B expression was suppressed (Fig 8C). Thus, UBE4B positively regulates Tax polyubiquitination, including K63-linked polyUb chains.

**Fig 9:**
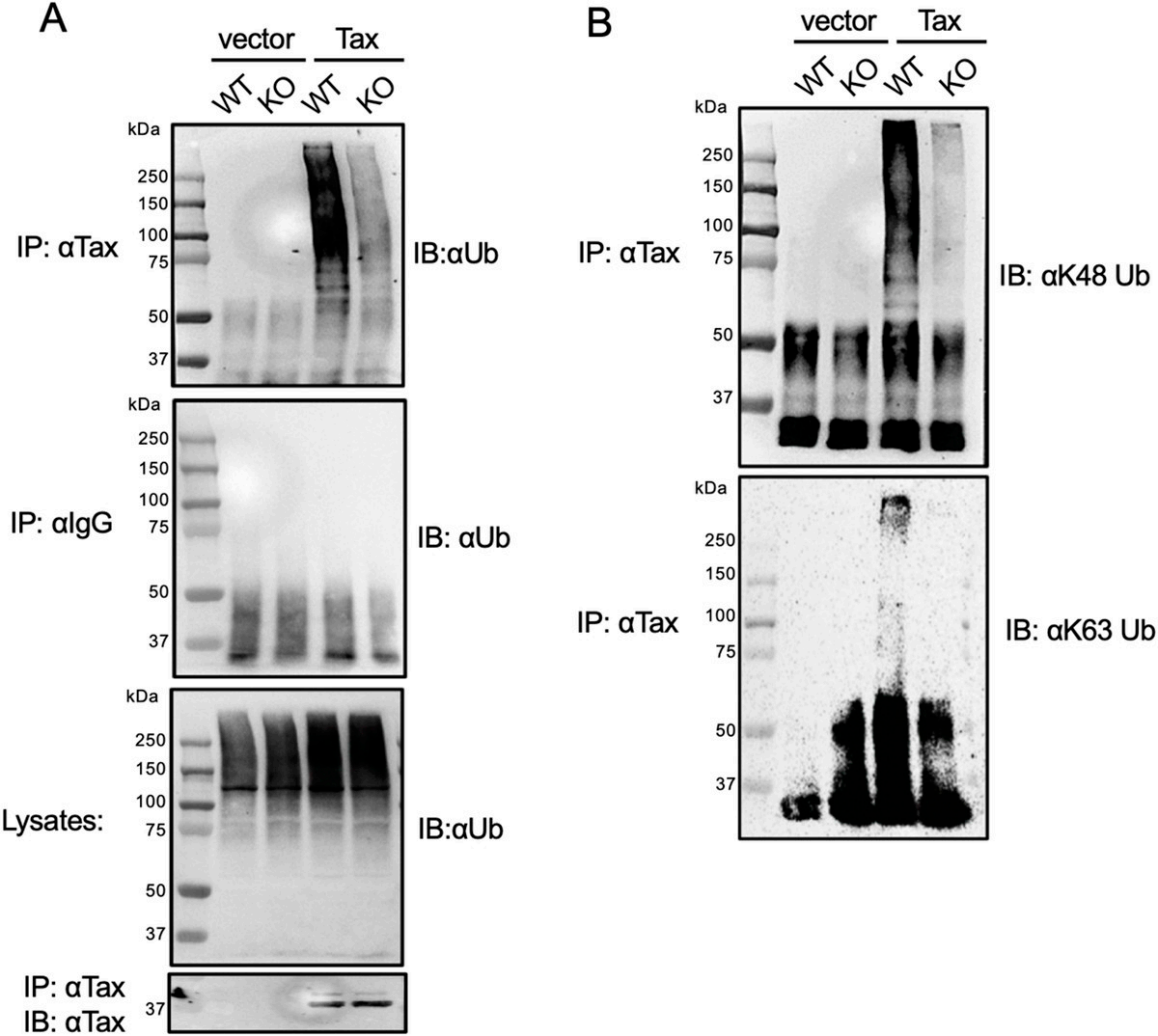
Tax polyubiquitination is impaired in UBE4B KO cells. (A-B) Ubiquitination assays were performed with Tax immunoprecipitates from lysates of wild-type and UBE4B KO (Clone H1) 293T cells transfected with Tax as indicated.

We further examined Tax polyUb in UBE4B KO 293T cells. Tax was expressed in control and UBE4B KO cells, and protein lysates were used for Tax ubiquitination assays. In agreement with our earlier shRNA knockdown experiments, Tax total and K63-linked polyUb chains were decreased in UBE4B KO cells (Fig 9A and 9B). We also found that Tax K48-linked polyUb chains were sharply reduced in UBE4B KO cells (Fig 9B). However, Tax stability, as determined by a cycloheximide chase assay, was similar in WT and UBE4B KO cells (S8 Fig). Therefore, UBE4B does not appear to promote Tax degradation, but is critical for both K48- and K63-linked Tax polyUb chains.

**Fig 10:**
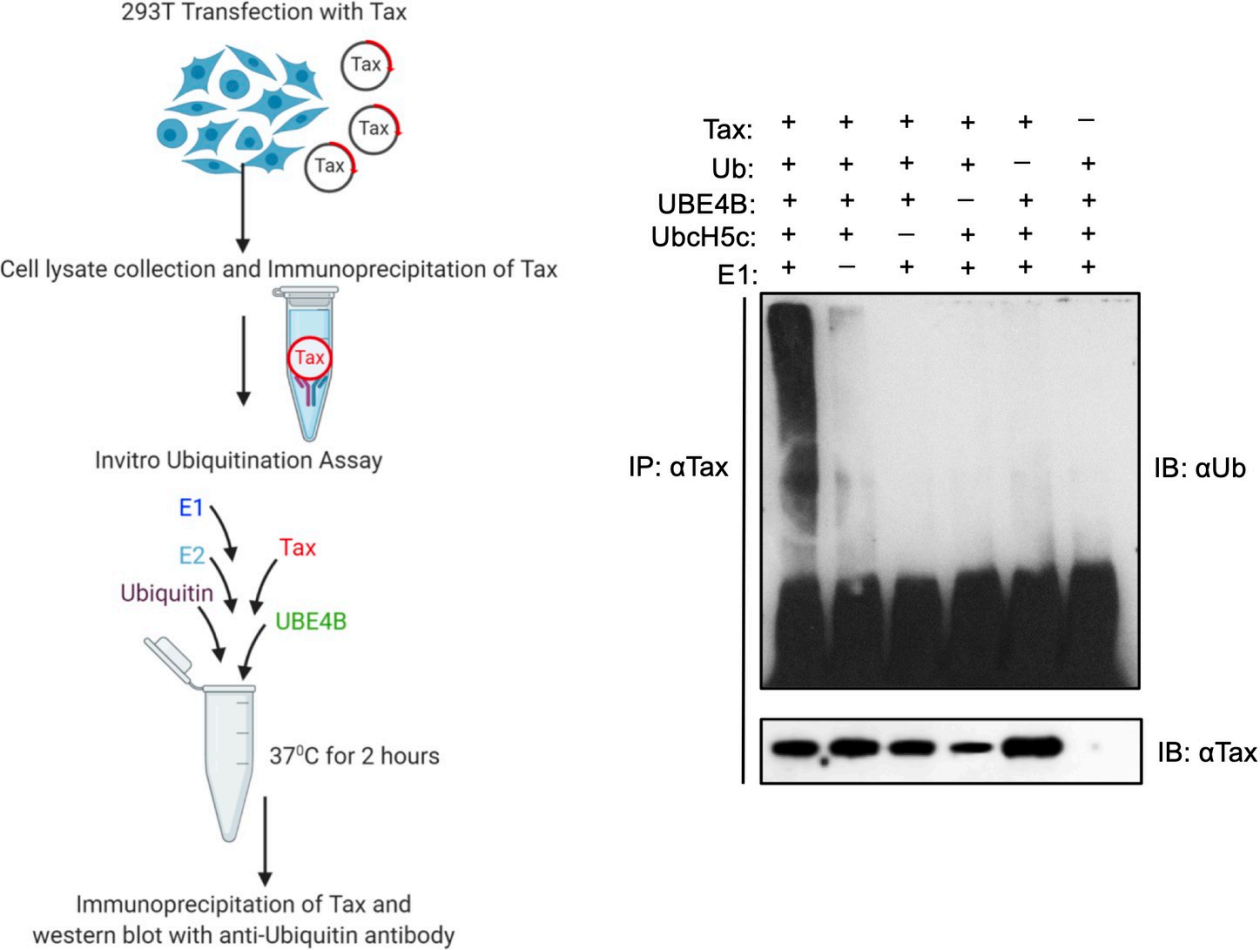
UBE4B directly ubiquitinates Tax. Schematic depicting the *in vitro* Tax ubiquitination assay protocol (left panel). Schematic was created with BioRender. *In vitro* ubiquitination assay was performed with the indicated recombinant proteins and immunoprecipitated Tax from lysates of transfected 293T cells. Following the Ub assay, Tax was immunoprecipitated from the reaction mixtures and immunoblotted with the indicated antibodies (right panel).

Since UBE4B and Tax interact, and UBE4B supports Tax polyubiquitination, we next determined if UBE4B could directly ubiquitinate Tax. To this end, we performed an *in vitro* ubiquitination assay with recombinant E1, E2 (UbcH5c), Ub and UBE4B-His6 proteins as well as immunopurified Tax from 293T cell lysates. After the *in vitro* Ub assay, Tax was subjected to a second IP followed by immunoblotting with anti-Ub. Tax polyUb chains were observed only in the presence of E1, E2, UBE4B, Ub and Tax (Fig 10). Therefore, we conclude that UBE4B can directly conjugate Tax with polyUb chains.

## Discussion

**Fig 11:**
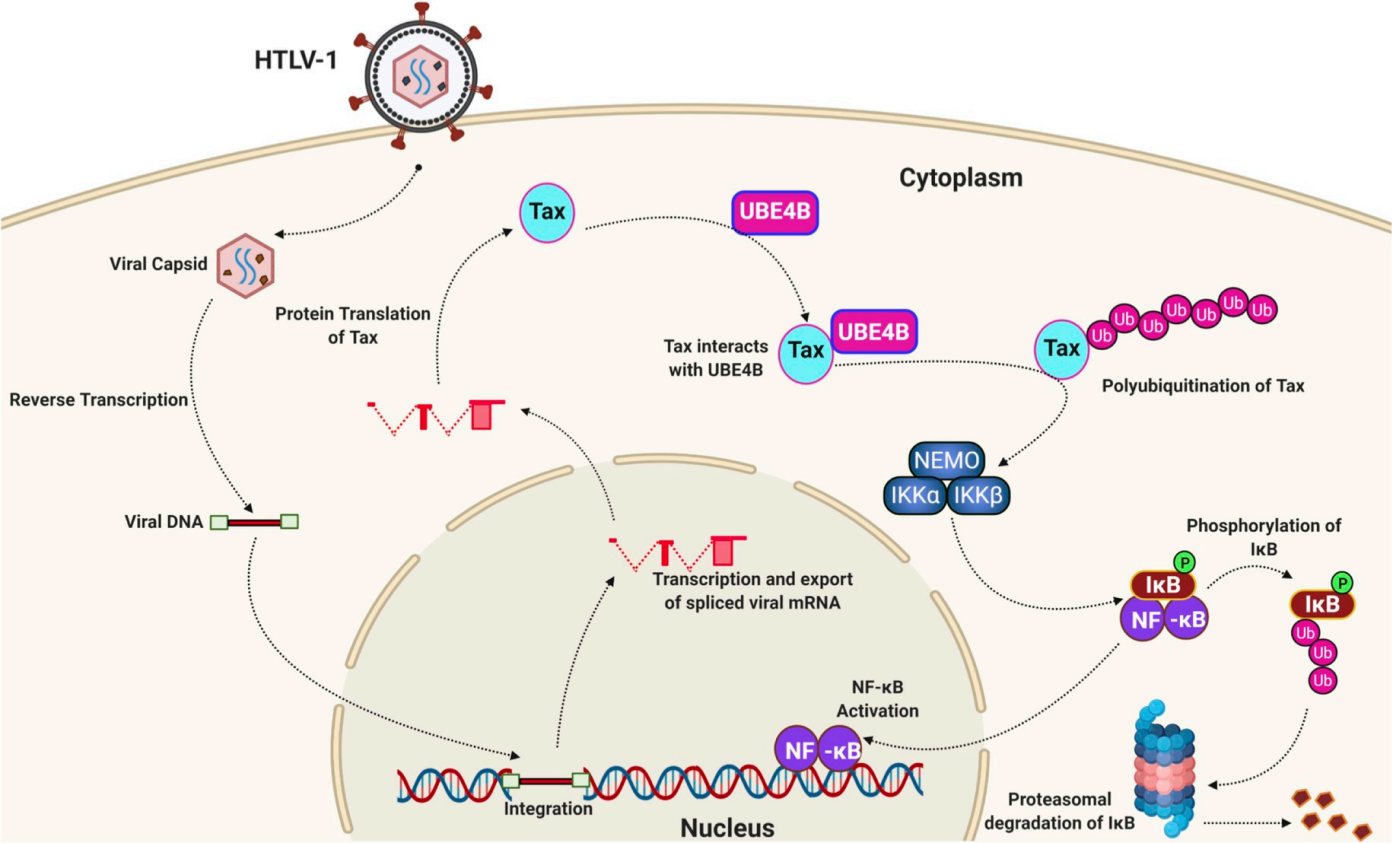
Model of UBE4B regulation of Tax polyubiquitination and NF-κB activation. Tax interacts with UBE4B, and UBE4B promotes Tax polyUb for downstream IKK and NF-κB activation. Created with BioRender.

Our study has identified the E3/E4 ubiquitin conjugation factor UBE4B as a novel interacting protein of HTLV-1 Tax. We have confirmed using multiple experimental approaches that UBE4B interacts with and colocalizes with Tax in HTLV-1-transformed T cells. Knockdown or knockout of UBE4B impairs Tax-induced NF-κB activation, as well as NF-κB signaling and cell survival in Tax+ HTLV-1-transformed cells. Furthermore, overexpression of UBE4B enhances Tax polyubiquitination, whereas loss of UBE4B impairs Tax K48- and K63-linked polyUb chains. Finally, we have demonstrated that UBE4B directly conjugates Tax with polyUb chains. Collectively, these results support the model depicted in Fig 11 whereby UBE4B interacts with and ubiquitinates Tax to activate IKK and canonical NF-κB signaling.

Our confocal microscopy and PLA experiments suggest that UBE4B and Tax mainly co-localize in a perinuclear region of the cytoplasm. Tax can localize within lipid rafts near the cis-Golgi where it directs the relocalization and activation of the IKK complex [19,50]. Since Tax K63-linked polyubiquitination is necessary to relocalize IKK to the cis-Golgi, UBE4B may play a role in the recruitment of IKK to the cis-Golgi. Tax-induced IKK activation and IκBα phosphorylation were impaired in UBE4B knockdown and knockout cells (Figs 4 and 6), consistent with UBE4B positioned upstream of the IKK complex together with Tax. Surprisingly, UBE4B was dispensable for noncanonical NF-κB activation in HTLV-1-transformed cells (Fig 4); the basis for UBE4B selectivity for canonical versus noncanonical NF-κB activation by Tax will need to be explored in future studies. Furthermore, UBE4B appears to selectively mediate Tax-induced NF-κB activation, but not TNF-triggered NF-κB suggesting a specific role of UBE4B in the regulation of Tax.

Knockdown of UBE4B triggered apoptotic cell death and diminished the proliferation of HTLV-1-transformed cell lines (Fig 7). Since UBE4B promotes Hdm2-mediated p53 proteasomal degradation in the nervous system [38], and Tax induces Hdm2-mediated Rb ubiquitination and subsequent degradation [49] we examined a potential regulation of p53 and Rb by UBE4B in HTLV-1-transformed T cells. However, knockdown of UBE4B had no influence on p53 and Rb protein expression in HTLV-1-transformed cell lines (S7 Fig). Therefore, it appears that the effects of UBE4B on the proliferation and survival of HTLV-1-transformed cells are mediated predominantly through NF-κB signaling. However, we cannot rule out additional effects of UBE4B on the cell cycle, ER stress/ERAD, or other pathways that impinge on cell proliferation and survival.

UBE4B supports both K63- and K48-linked polyubiquitination of Tax (Figs 8 and 9). Although it has not been demonstrated that UBE4B can catalyze K63-linked polyUb chains, this does not rule out the possibility that UBE4B conjugates Tax with K63-linked polyUb chains. The specificity of the polyUb linkage catalyzed by a U-box ligase is determined by the E2 ubiquitin-conjugating enzyme, rather than the E3 enzyme. Furthermore, UBE4B can utilize UbcH5c as an E2 enzyme, which has the potential to catalyze K63-linked polyUb chains [51]. Our *in vitro* ubiquitination assay (Fig 10) supports the notion that UBE4B directly ubiquitinates Tax; however, we cannot distinguish if the E3 or E4 activity of UBE4B contributes to Tax polyubiquitination. Immunopurified Tax from cell lysates likely has post-translational modifications including mono- and polyUb which could potentially be extended and fine-tuned by the E4 activity of UBE4B. Therefore, we envision two potential mechanisms of UBE4B regulation of Tax polyubiquitination: 1) UBE4B functions as an E3 ligase for Tax and conjugates Tax with both K48- and K63-linked polyUb chains, or 2) UBE4B functions as an E4 enzyme and extends K48- and/or K63-linked polyUb chains primed by other E3 enzymes. Tax itself has been proposed to have E3 ligase activity [52], however we did not observe Tax polyUb chains in the absence of UBE4B in an *in vitro* Ub assay (Fig 10).

It is curious that UBE4B promotes K48-linked polyubiquitination of Tax but does not appear to trigger its degradation. Based on these findings, one possibility is that UBE4B may mediate branched K48-K63 polyUb chains on Tax. In this regard, it has been reported that the E3 ligase TRAF6 is conjugated with branched K48-K63 polyUb chains by the E3 ubiquitin ligase HUWE1, and these branched chains enhance TRAF6-mediated NF-κB activation by inhibiting the disassembly of K63-linked polyUb chains by deubiquitinating enzymes such as CYLD [53]. Similarly, UBE4B may potentially catalyze branched K48-K63 polyUb chains on Tax to stabilize and protect the K63-linked polyUb chains from negative regulators, and therefore potentiate persistent NF-κB signaling. There is precedence for E4 enzymes in synthesizing branched Ub chains as yeast Ufd2p can catalyze K29-K48 branched polyUb chains to promote degradation of substrates [54]. Additional experiments are needed to further investigate if Tax is conjugated with branched K48-K63 polyUb chains, and if UBE4B is implicated in this process.

Overall, we have identified the E3/E4 conjugation factor UBE4B as a Tax binding partner that promotes Tax polyubiquitination and canonical NF-κB activation. Since Tax K63-linked polyubiquitination is required for constitutive NF-κB activation and subsequent immortalization and transformation of CD4+ T cells, UBE4B may represent a novel therapeutic target for Tax+ ATLL tumors (~40% of ATLL tumors express Tax sporadically).

## Materials and methods

### Ethics statement

Whole blood from a healthy donor was purchased from Biological Specialty Corporation (Colmar, PA). Written informed consent was obtained from the subject and the sample was de-identified by the company prior to our receipt.

### Reagents, plasmids and antibodies

Human embryonic kidney cells (HEK 293T) were purchased from ATCC. PBMCs were isolated from whole blood as described previously [55]. Cell lines Jurkat, Jurkat Tax Tet-on, C8166, MT-2, MT-4, HUT-102, TL-OM1, ED40515(-) and ATL43T were described previously [55,56]. HEK 293T cells were cultured in Dulbecco’s modified Eagle’s medium (DMEM); PBMCs, Jurkat, Jurkat Tax Tet-on, C8166, MT-2, MT-4, HUT-102, TL-OM1, ED40515(-) and ATL43T cells were cultured in RPMI medium. Medium was supplemented with fetal bovine serum (10%) and penicillin-streptomycin (1%). Expression vectors encoding Flag-Tax, pCMV4-Tax, Tax M22, Tax M47, HTLV-1 LTR-Luc, NF-κB-TATA Luc, pRL-tk, pDUET-Tax, psPAX2 and VSV-G were described previously [22,31,57]. pDEST51-UBE4B-Flag was a gift from Dr. Sarah Spinette [58]. Site-directed mutagenesis of UBE4B (P1140A) was generated by PCR using Platinum Pfx DNA polymerase (Thermo Fisher Scientific). The monoclonal anti-Tax antibody was prepared from a Tax hybridoma (168B17-46-34) received from the AIDS Research and Reference Program, NIAID, National Institutes of Health. Anti-Tax antibody (1A3) was purchased from Santa Cruz Biotechnology. Alexa Fluor 594-conjugated donkey anti-mouse IgG and Alexa Fluor 488-conjugated donkey anti-rabbit IgG were purchased from Thermo Fisher Scientific. The monoclonal Flag M2 and hemagglutinin (HA; 12CA5) antibodies were purchased from Millipore-Sigma. UBE4B antibodies were purchased from Bethyl Laboratories and Santa Cruz Biotechnology. The NEMO, LDH, Rb, p53, c-Rel and caspase 3 antibodies were from Santa Cruz Biotechnology. PARP, cleaved PARP, cleaved caspase 3, pIκBα, pIKKα/β, p105/50, p100/p52, IKKβ, IκBα, Vinculin, β-Actin, K48- and K63 linkage-specific Ub antibodies were from Cell Signaling Technology. Ubiquitin antibody was purchased from Enzo Life Sciences. DAPI (4’, 6-diamidino-2-phenylindole) was purchased from EMD Biosciences. TNF was purchased from R&D Systems. Cycloheximide solution was purchased from Millipore-Sigma. Doxycycline and Tet-free FBS were purchased from Takara and puromycin from Thermo Fisher Scientific.

### Transfections and luciferase reporter assays

293T cells were transiently transfected with GenJet In Vitro DNA Transfection Reagent (SignaGen Laboratories). Luciferase reporter assays were performed 24 h after DNA transfection, unless otherwise indicated, using the Dual-Glo luciferase assay system (Promega). Firefly luciferase values were normalized based on the *Renilla* luciferase internal control values. Luciferase values are presented as “fold induction” compared to the control transfected with empty vector.

### Immunoblotting, co-immunoprecipitation and ubiquitination assays

Whole cell lysates were generated by lysing cells in RIPA buffer (50 mM Tris-Cl [pH 7.4], 150 mM NaCl, 1% NP-40, 0.25% sodium deoxycholate, 1 mM phenylmethylsulfonyl fluoride [PMSF], 1× Roche complete mini-protease inhibitor cocktail) on ice, followed by centrifugation. Cell lysates were resolved by SDS-PAGE, transferred to nitrocellulose membranes, and subjected to immunoblotting with the indicated primary antibodies and HRP-conjugated secondary antibodies (GE Healthcare Life Sciences). Immunoreactive bands were visualized by Western Lightning enhanced chemiluminescence (PerkinElmer). For co-IPs, lysates were diluted 1:1 in RIPA buffer and precleared with protein A agarose beads (Millipore-Sigma) for 60 min at 4°C. Pre-cleared lysates were further incubated at 4°C overnight with the indicated antibodies (1 to 3 μl) and protein A agarose or protein G Dynabeads (Thermo Fisher Scientific). Immunoprecipitates were washed three times with RIPA buffer (LSB) to elute bound proteins. An additional wash with RIPA buffer supplemented with 1 M urea was performed for ubiquitination assays. For Tax ubiquitination assays performed with 6xHis-Tax, cells were lysed in buffer B (100 mM NaH_2_PO_4_, 10 mM Tris, and 8 M urea [pH 8.0]) and His-tagged Tax proteins were precipitated with Ni-nitrilotriacetic acid (NTA) agarose (Qiagen). After washing in buffer C (100 mM NaH_2_PO_4_, 10 mM Tris, and 8 M urea [pH 6.3]), His-tagged proteins were eluted in buffer E (100 mM NaH_2_PO_4_, 10 mM Tris, and 8 M urea [pH 4.5]) and subjected to SDS-PAGE and immunoblotting.

### EMSA DNA binding assay

Nonradioactive EMSA was performed as described previously [55] using LightShift Chemiluminescent EMSA Kit (Thermo Scientific) according to the manufacturer’s instructions.

### Confocal microscopy

MT-2 and C8166 cells were cultured for 4 h on glass coverslips coated with poly-L-lysine in 6-well plates. Cells were fixed with 4% paraformaldehyde for 30 min and permeabilized with 0.5% Triton X-100 for 5 min. The fixed cells were then incubated with 5% BSA for 1 h followed by staining with mouse anti-Tax or rabbit anti-UBE4B antibodies overnight at 4°C. Coverslips were incubated with Alexa Fluor 594-conjugated donkey anti-mouse IgG, Alexa Fluor 488-conjugated donkey anti-rabbit IgG (Thermo Fisher Scientific), and DAPI to stain nuclei. Images were acquired with a C2+ confocal microscope system (Nikon), and processed using NIS Elements software.

### Proximity ligation assay

PLA was performed with the DuoLink In Situ Red Starter Kit Mouse/Rabbit (Millipore-Sigma) as recommended by the manufacturer. MT-2 and C8166 cells were grown on glass coverslips, fixed, permeabilized and incubated with primary antibodies: anti-UBE4B (Rabbit) and anti-Tax (Mouse). Slides were incubated with Duolink PLA probes, ligated, amplified and washed. Images were acquired with a C2+ confocal microscope system (Nikon). PLA signals were quantified using ImageJ software.

### *In vitro* ubiquitination assay

Recombinant E1 ubiquitin activating enzyme, E2 (UbcH5c), Ub and UBE4B-His6 were purchased from BostonBiochem/R&D Systems. Tax was transfected into 293T cells and lysates immunoprecipitated with anti-Tax. Ub assay was performed on eluted immunoprecipitated Tax after addition of E1, UbcH5c and UBE4B-His6, and ubiquitin conjugation reaction buffer for 2 h at 37°C. Tax was re-immunoprecipitated with anti-Tax followed by immunoblotting with anti-Ub.

### Quantitative real-time PCR (qRT-PCR)

RNA was isolated using the RNeasy minikit (Qiagen). RNA was converted to cDNA using the First Strand cDNA synthesis kit for reverse transcription (avian myeloblastosis virus [AMV]; Millipore-Sigma). Quantitative real-time PCR (qRT-PCR) was performed with an Applied Biosystems 7500 Real-Time PCR system using KiCqStart SYBR Green qPCR ReadyMix (Millipore-Sigma). Gene expression was normalized to the internal control 18S rRNA. Primer sequences for qRT-PCR are provided in S1 Table.

### CHX chase assay

Cycloheximide (CHX) chase assays were performed as described previously [31]. Cells were treated with CHX (10 μg/ml) for various times 2 days after transfection. Cells were lysed in RIPA buffer, and immunoblotting was conducted with anti-Tax.

### Knockdown with lentiviral shRNAs

Three lentiviral Mission short hairpin RNA (shRNA) clones targeting UBE4B were obtained from Millipore-Sigma. HEK293T cells were transfected with the lentiviral shRNA-targeting vectors, psPAX2 packaging plasmid (Addgene) and vesicular stomatitis virus glycoprotein (VSV-G). After 48 h, the supernatants were collected and centrifuged at 25,000 rpm using a Beckman SW28 centrifuge rotor for 2 h at 4°C. The supernatants were removed, and the pellets were re-suspended in ice-cold phosphate-buffered saline (PBS). Viral stocks were used to infect cell lines.

### Knockdown with inducible lentiviral shRNAs

Three SMARTvector human inducible lentiviral shRNA (piSMART mCMV/TurboGFP) plasmids targeting UBE4B were obtained from Horizon. Lenti-X 293T cells were transfected with Lenti-X packaging single shots (Takara) and lentiviral supernatants were collected 72 h post-transfection. The supernatants were centrifuged at 500 x g for 10 minutes to remove cell debris and concentrated with the Lenti-X concentrator (Takara). The concentrated lentiviral stocks were quantified with the Lenti-XTM qRT-PCR titration kit (Takara) and transduced (MOI = 25) in C8166, MT-2 and TL-OM1 cells. After 48 h, transduced cells were selected with 2 μg/ml of puromycin and treated with 0.5 μg/ml of Dox for four days after selection.

### CRISPR/Cas9 knockout

CRISPR/Cas9-mediated knockout of UBE4B was performed as previously described [59]. Briefly, gRNAs specific for UBE4B exon 10 were designed through the E-CRISP web server (http://www.e-crisp.org/E-CRISP/) and expressed by pLentiCRISPRv2-puro (a gift from Feng Zhang; Addgene). Guide sequence function was analyzed using the Surveyor Mutation Detection Kit (Integrated DNA Technologies) according to the manufacturer’s instructions. The genomic DNA fragments including gRNA target sequences were PCR-amplified and analyzed by agarose gel electrophoresis. Individual clones were isolated by limiting dilution and genomic DNA purified, *Ube4b* exon 10 amplified by PCR and subjected to Sanger DNA sequencing. Primer sequences for gRNAs and Surveyor primers are provided in S1 Table.

### Cell viability and proliferation assays

Cell viability was determined using the CellTiter-Glo luminescent cell viability assay (Promega), which quantitates ATP as a measure of metabolically active cells. A total of 50 μl of suspended cells and 50 μl of CellTiter-Glo solution were mixed and incubated at room temperature for 10 min, and the luminescence was quantified with a GloMax96 microplate luminometer (Promega).

### Yeast Two-Hybrid Analysis

Yeast two-hybrid screening was performed by Hybrigenics Services, S.A.S., Paris, France (http://www.hybrigenics-services.com). The coding sequence for the full length Tax protein was PCR-amplified and cloned into pB27 as a C-terminal fusion to LexA (N-LexA-Tax-C). The construct was verified by sequencing the entire insert and used as bait to screen a random-primed human leukocyte and activated mononuclear cells cDNA library constructed into pP6. pB27 and pP6 derive from the original pBTM116 [60] and pGADGH [61] plasmids, respectively. 46.7 million clones were screened using a mating approach with YHGX13 (Y187 ade2-101::loxP-kanMX-loxP, matα) and L40ΔGal4 (mata) yeast strains as previously described [62]. 282 His+ colonies were selected on a medium lacking tryptophan, leucine and histidine. The prey fragments of the positive clones were amplified by PCR and sequenced at their 5’ and 3’ junctions. The resulting sequences were used to identify the corresponding interacting proteins in the GenBank database (NCBI) using a fully automated procedure. A confidence score (PBS, for Predicted Biological Score) was attributed to each interaction as previously described [63].

### Statistical analysis

Data are expressed as mean fold increase ± standard deviation relative to the control from a representative experiment performed 3 times in triplicate. Statistical analysis was performed in GraphPad Prism 8, and indicated in the Figure legends and Supplemental Figure legends.

## Supporting information

S1 Fig Interaction of Tax and UBE4B mutants. (A-B) Co-IP analysis with Flag-UBE4B immunoprecipitates from lysates of 293T cells transfected with the indicated plasmids. Immunoblotting was performed with lysates using the indicated antibodies.
(TIF)

S2 Fig Specificity of Tax-UBE4B colocalization. (A) Immunofluorescence confocal microscopy was performed using Jurkat cells with the indicated antibodies. (B) PLA was performed using Jurkat cells with Tax and UBE4B antibodies.
(TIF)

S3 Fig Validation of UBE4B shRNAs. (A) Immunoblotting was performed with anti-UBE4B using whole cell lysates from 293T cells expressing either Mission control scrambled shRNA or UBE4B shRNAs 1–3. (B) Immunoblotting was performed with anti-UBE4B using lysates from 293T cells expressing SMARTvector human inducible lentiviral plasmids with UBE4B shRNAs 1–3 and treated with Dox. (C) Incucyte S3 live-cell analysis of GFP expression using 293T cells expressing SMARTvector human inducible lentiviral plasmids with UBE4B shRNAs 1–3 and treated with Dox. (D) Fluorescence microscopy was performed using a Nikon DS-Fi3 Microscope camera with MT-2, C8166 and TL-OM1 cells stably expressing SMARTvector inducible lentiviral plasmid with UBE4B shRNA #2 and treated with Dox.
(TIF)

S4 Fig UBE4B does not interact with NEMO. Co-IP analysis with either control IgG, anti-NEMO or anti-UBE4B immunoprecipitates from lysates of C8166 and MT-2 cells as indicated.
(TIF)

S5 Fig Tax does not upregulate the expression of UBE4B. (A) qRT-PCR of Tax, CD25 and UBE4B mRNAs in Jurkat Tax Tet-on cells treated either with Dox or DMSO. (B) qRT-PCR of UBE4B mRNA in Jurkat, ATLL cell lines, and PBMCs. (C) Immunoblotting was performed with the indicated antibodies using whole cell lysates from Jurkat, Tax+ and Tax- ATLL cell lines. Unpaired Student’s *t*-test, **P <0.01, ***P value of <0.001, ns = not significant.
(TIF)

S6 Fig Characterization of UBE4B knockout 293T clones. (A) DNA sequencing chromatograms of PCR-amplified UBE4B exon 10 from genomic DNA derived from wild-type (E2, H10) and UBE4B KO (G12, H1, F5) 293T cell clones. UBE4B KO clones G12 and H1 both have an adenine insertion. (B) Immunoblotting was performed with the indicated antibodies using lysates from wild-type (E2, H10) and UBE4B KO (G12, H1, F5) 293T cell clones.
(TIF)

S7 Fig UBE4B does not promote Rb and p53 degradation in HTLV-1-transformed cell lines. Immunoblotting was performed with the indicated antibodies using lysates from Jurkat, MT-2, C8166 and HUT-102 cells expressing control or UBE4B shRNAs.
(TIF)

S8 Fig UBE4B does not destabilize Tax. CHX chase assay with lysates from wild-type and UBE4B KO 293T cells (clone H1) transfected with Tax and treated with cycloheximide for the indicated times. Immunoblotting was performed with the indicated antibodies.
(TIF)

S1 Table Oligonucleotides used in the study. (PDF)

S1 Movie Tax-UBE4B colocalization in C8166 cells. 3D projection and rotation around the X axis using confocal microscopy depicting the localization and interaction of Tax and UBE4B in C8166 cells. Tax was detected with Alexa 488 and UBE4B detected with Alexa 594.
(M4V)

S2 Movie Tax-UBE4B colocalization in MT-2 cells. 3D projection and rotation around the X axis using confocal microscopy depicting the localization and interaction of Tax and UBE4B in MT-2 cells. Tax was detected with Alexa 488 and UBE4B detected with Alexa 594.
(M4V)
