# Supplementary figures and images for: The E3/E4 ubiquitin conjugation factor UBE4B interacts with and ubiquitinates the HTLV-1 Tax oncoprotein to promote NF-κB activation
Source: PLoS Pathog. 2020 Dec 23;16(12):e1008504. doi: 10.1371/journal.ppat.1008504 (PMC7790423; doi:10.1371/journal.ppat.1008504)

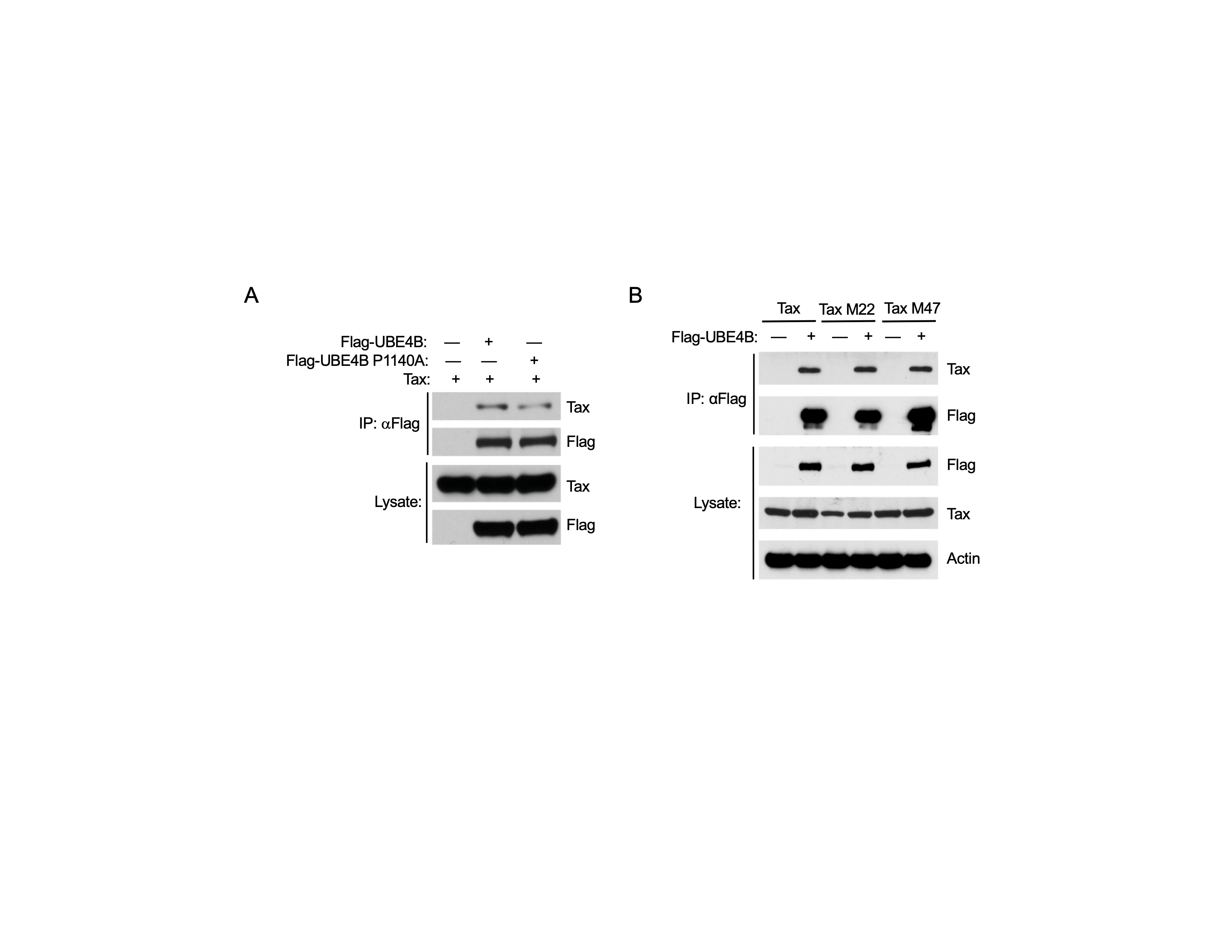

Supplement: S1 Fig — (A-B) Co-IP analysis with Flag-UBE4B immunoprecipitates from lysates of 293T cells transfected with the indicated plasmids. Immunoblotting was performed with lysates using the indicated antibodies. (TIF) [file ppat.1008504.s001.tif]

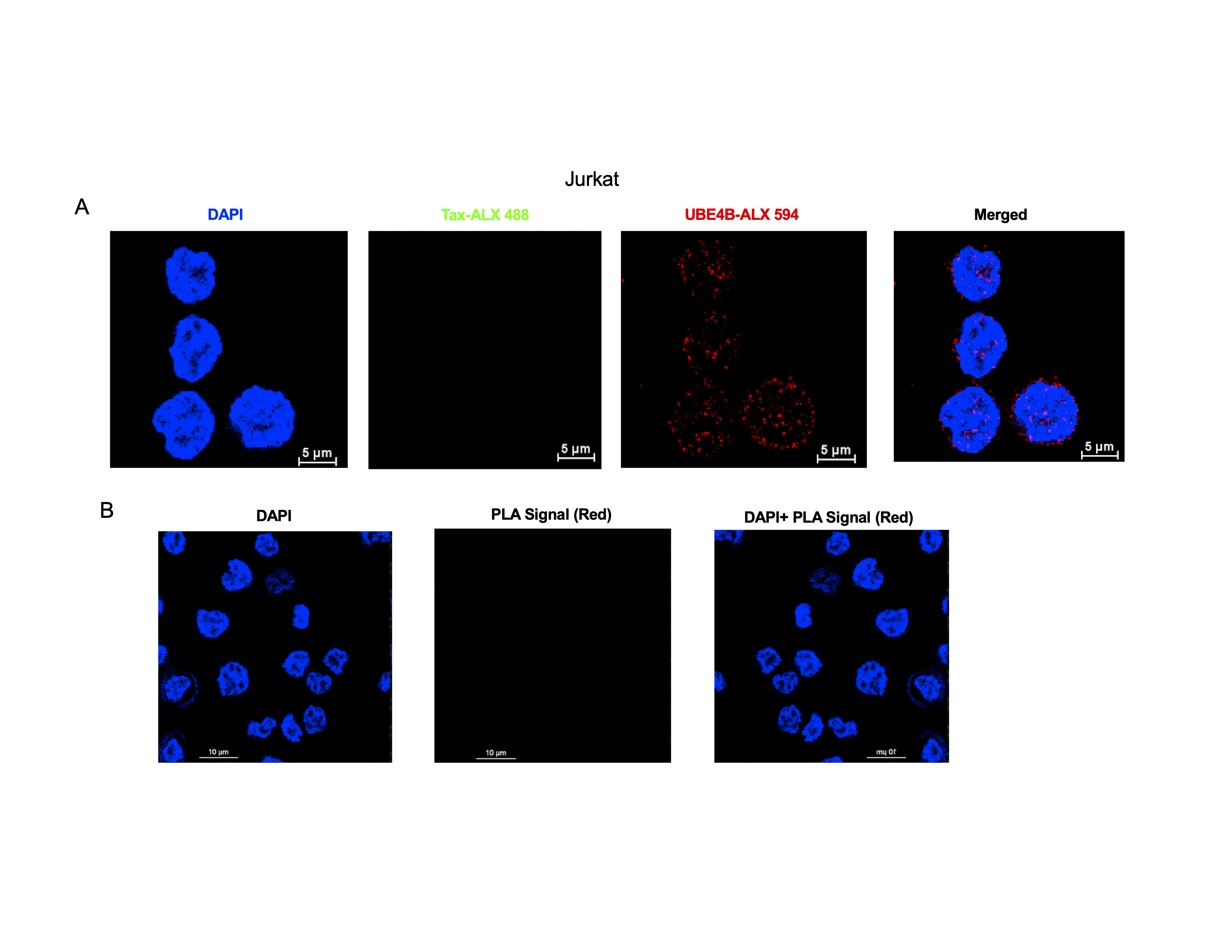

Supplement: S2 Fig — (A) Immunofluorescence confocal microscopy was performed using Jurkat cells with the indicated antibodies. (B) PLA was performed using Jurkat cells with Tax and UBE4B antibodies. (TIF) [file ppat.1008504.s002.tif]

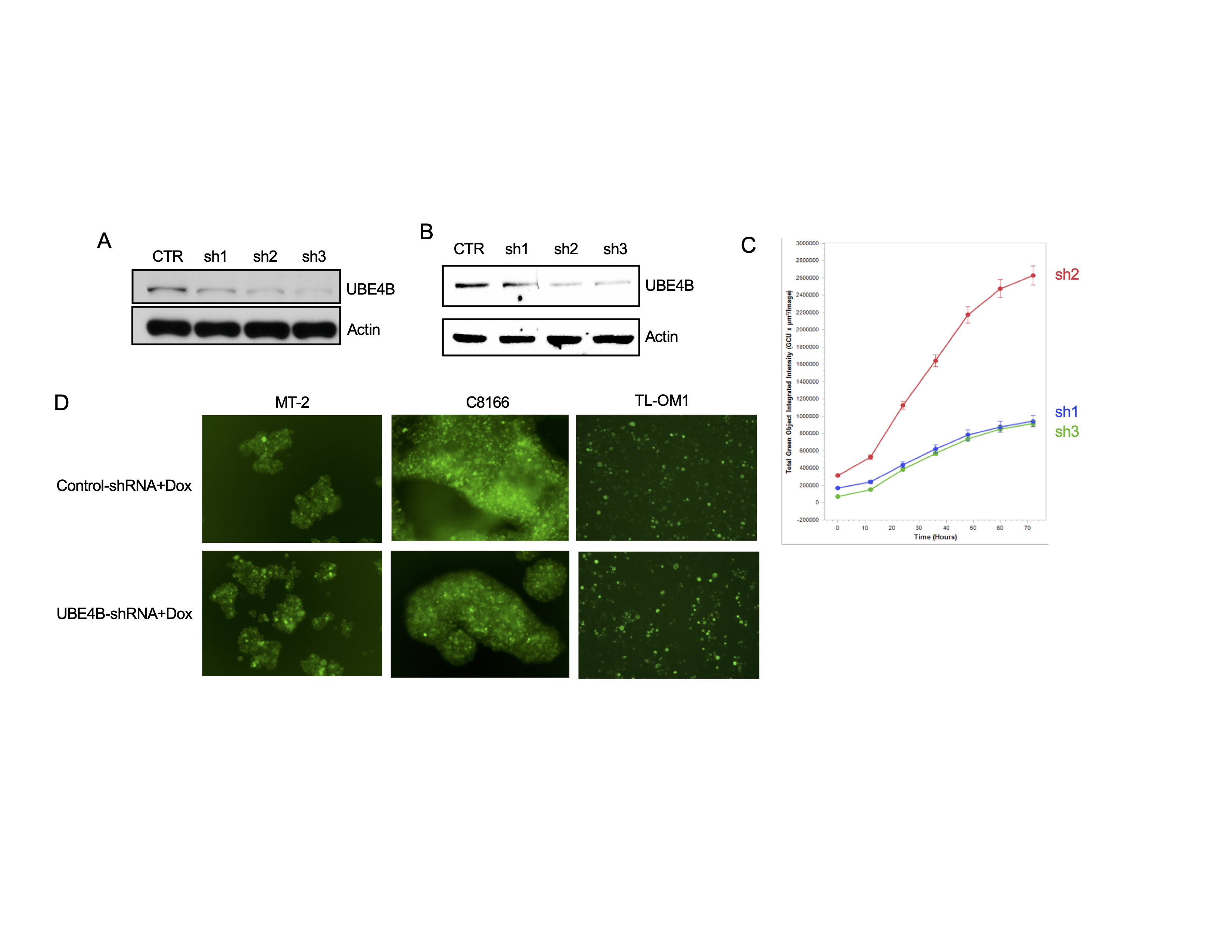

Supplement: S3 Fig — (A) Immunoblotting was performed with anti-UBE4B using whole cell lysates from 293T cells expressing either Mission control scrambled shRNA or UBE4B shRNAs 1–3. (B) Immunoblotting was performed with anti-UBE4B using lysates from 293T cells expressing SMARTvector human inducible lentiviral plasmids with UBE4B shRNAs 1–3 and treated with Dox. (C) Incucyte S3 live-cell analysis of GFP expression using 293T cells expressing SMARTvector human inducible lentiviral plasmids with UBE4B shRNAs 1–3 and treated with Dox. (D) Fluorescence microscopy was performed using a Nikon DS-Fi3 Microscope camera with MT-2, C8166 and TL-OM1 cells stably expressing SMARTvector inducible lentiviral plasmid with UBE4B shRNA #2 and treated with Dox. (TIF) [file ppat.1008504.s003.tif]

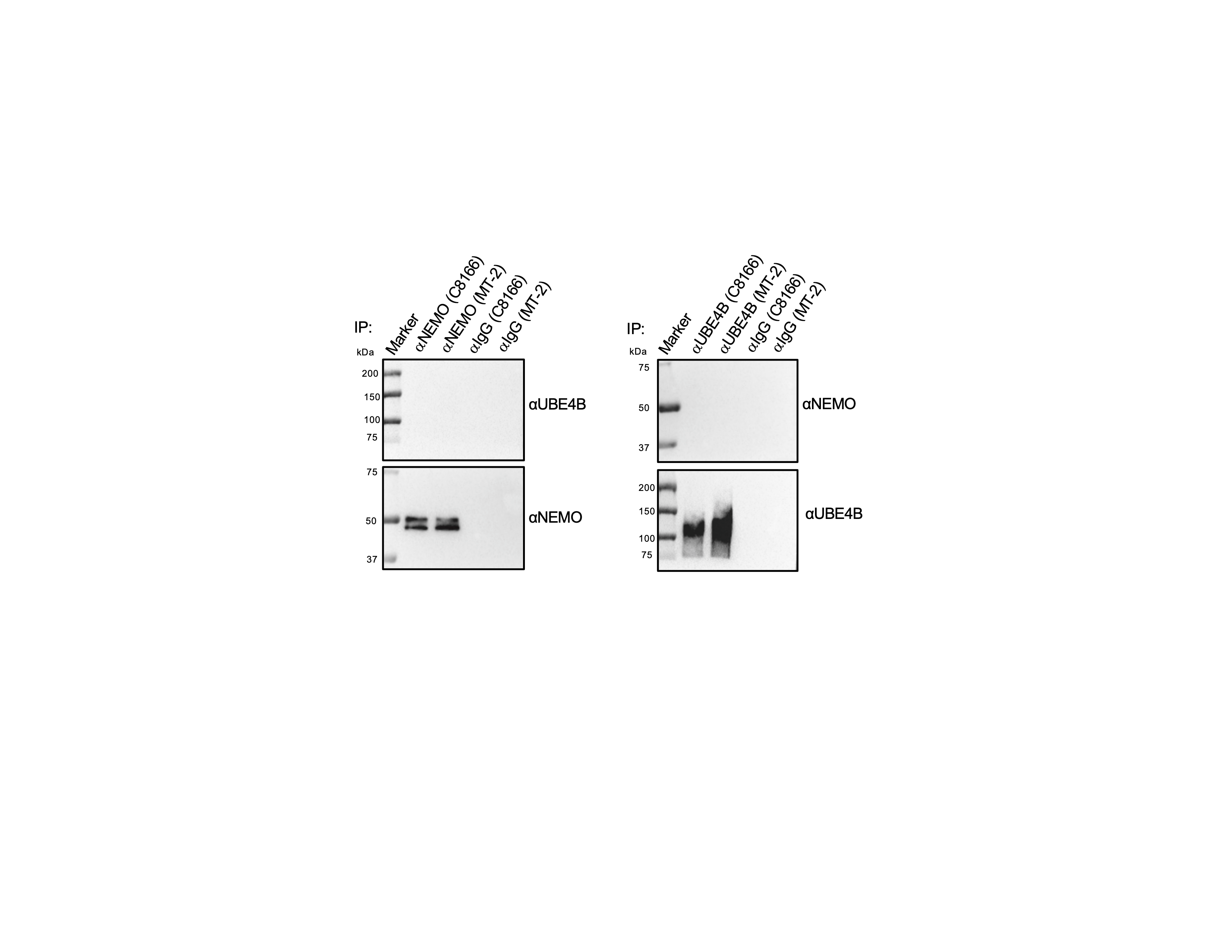

Supplement: S4 Fig — Co-IP analysis with either control IgG, anti-NEMO or anti-UBE4B immunoprecipitates from lysates of C8166 and MT-2 cells as indicated. (TIF) [file ppat.1008504.s004.tif]

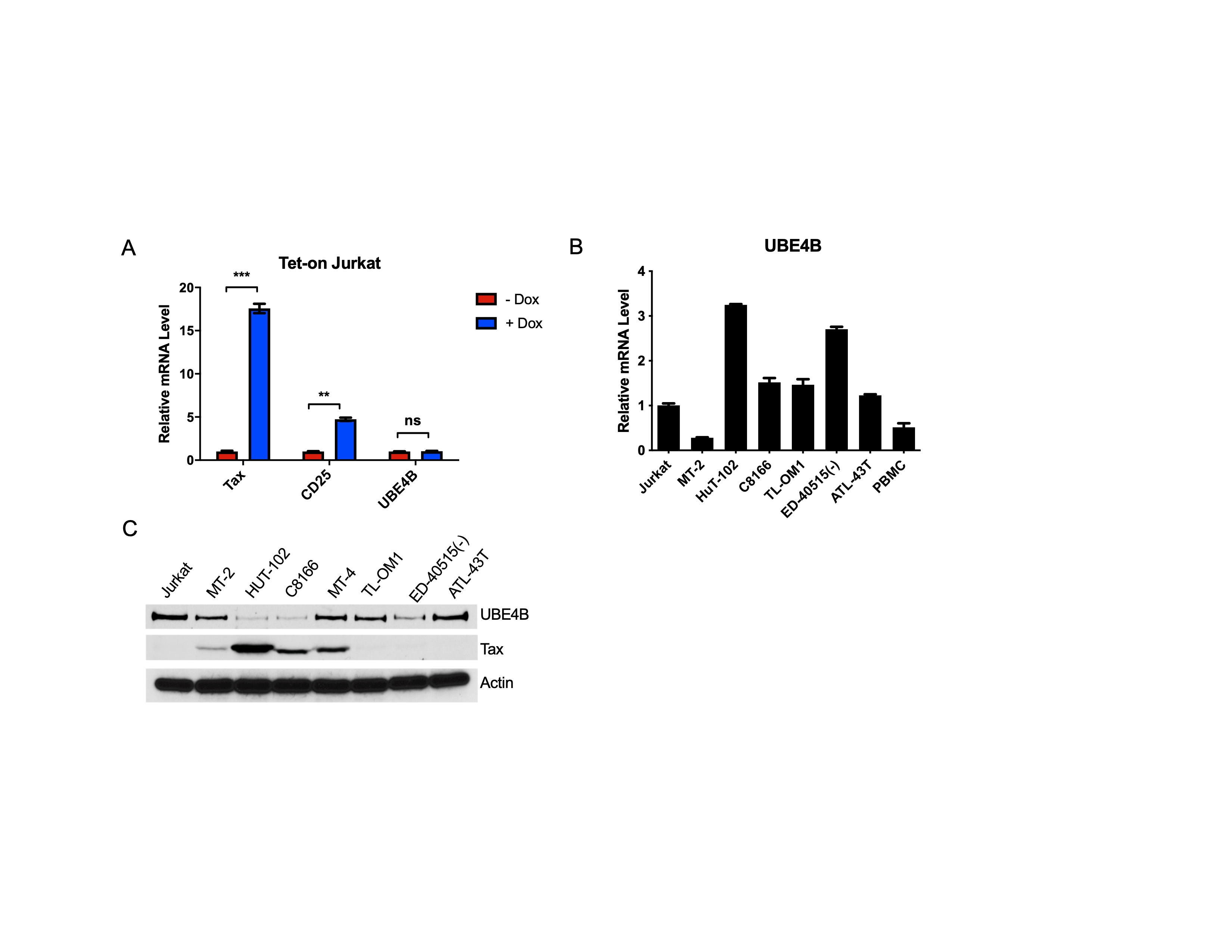

Supplement: S5 Fig — (A) qRT-PCR of Tax, CD25 and UBE4B mRNAs in Jurkat Tax Tet-on cells treated either with Dox or DMSO. (B) qRT-PCR of UBE4B mRNA in Jurkat, ATLL cell lines, and PBMCs. (C) Immunoblotting was performed with the indicated antibodies using whole cell lysates from Jurkat, Tax+ and Tax- ATLL cell lines. Unpaired Student’s t-test, **P <0.01, ***P value of <0.001, ns = not significant. (TIF) [file ppat.1008504.s005.tif]

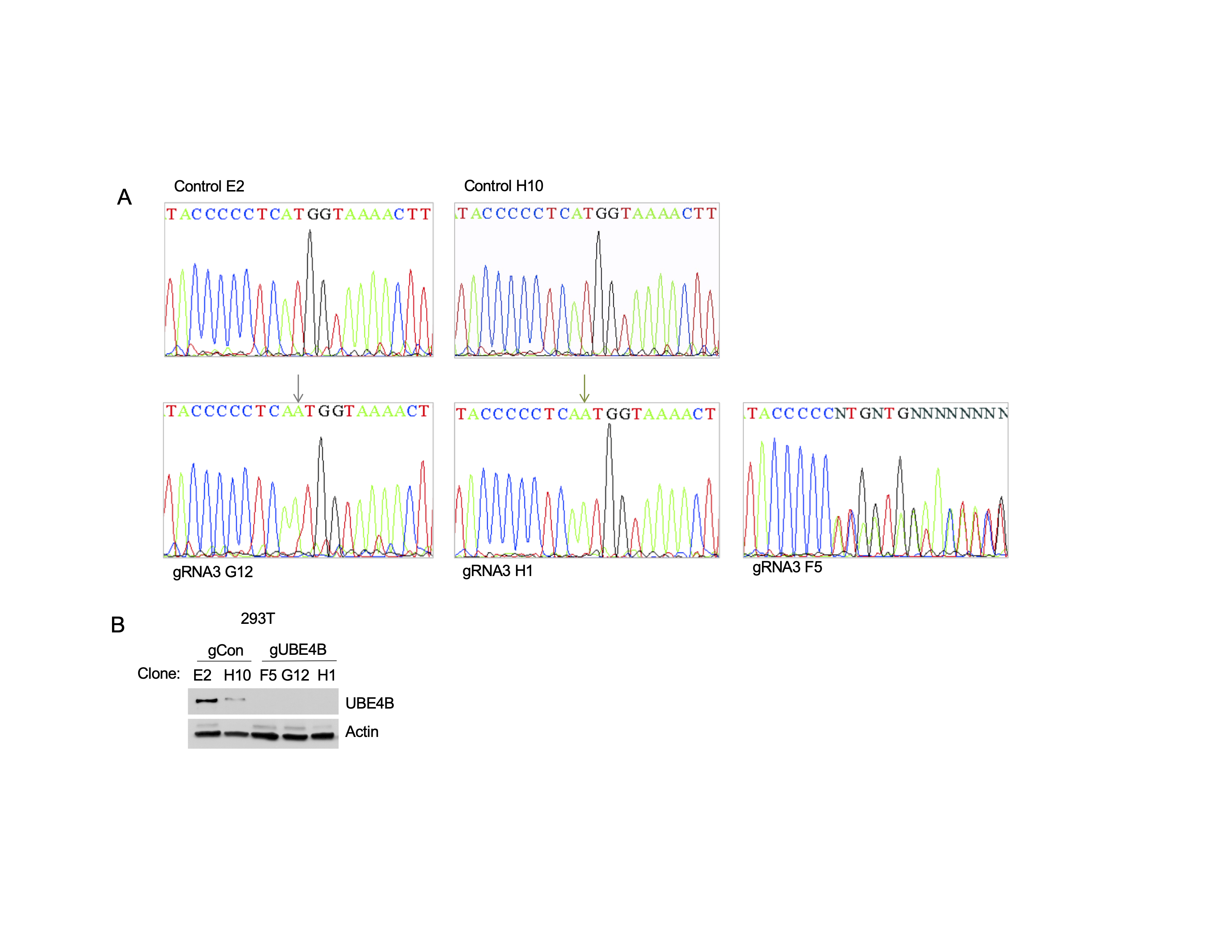

Supplement: S6 Fig — (A) DNA sequencing chromatograms of PCR-amplified UBE4B exon 10 from genomic DNA derived from wild-type (E2, H10) and UBE4B KO (G12, H1, F5) 293T cell clones. UBE4B KO clones G12 and H1 both have an adenine insertion. (B) Immunoblotting was performed with the indicated antibodies using lysates from wild-type (E2, H10) and UBE4B KO (G12, H1, F5) 293T cell clones. (TIF) [file ppat.1008504.s006.tif]

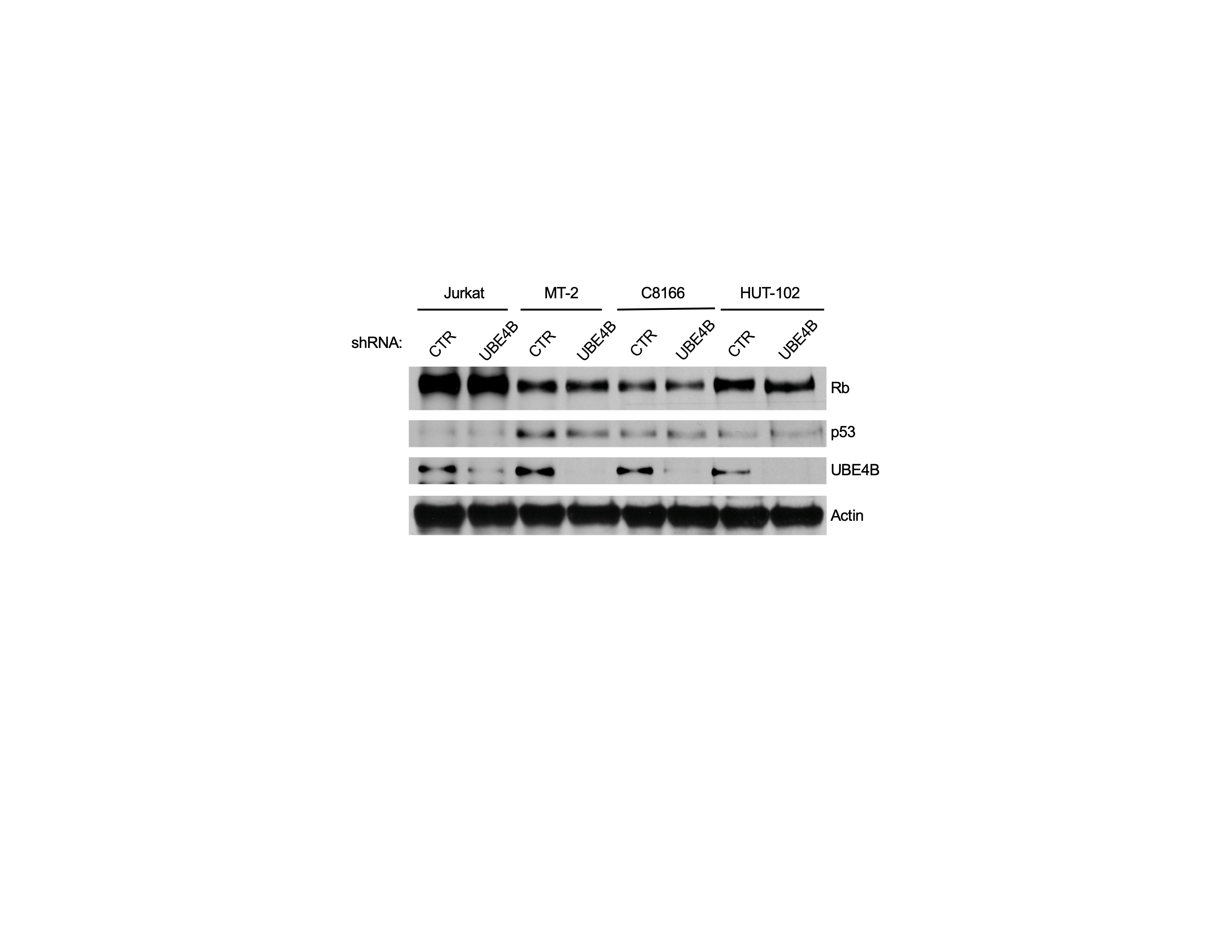

Supplement: S7 Fig — Immunoblotting was performed with the indicated antibodies using lysates from Jurkat, MT-2, C8166 and HUT-102 cells expressing control or UBE4B shRNAs. (TIF) [file ppat.1008504.s007.tif]

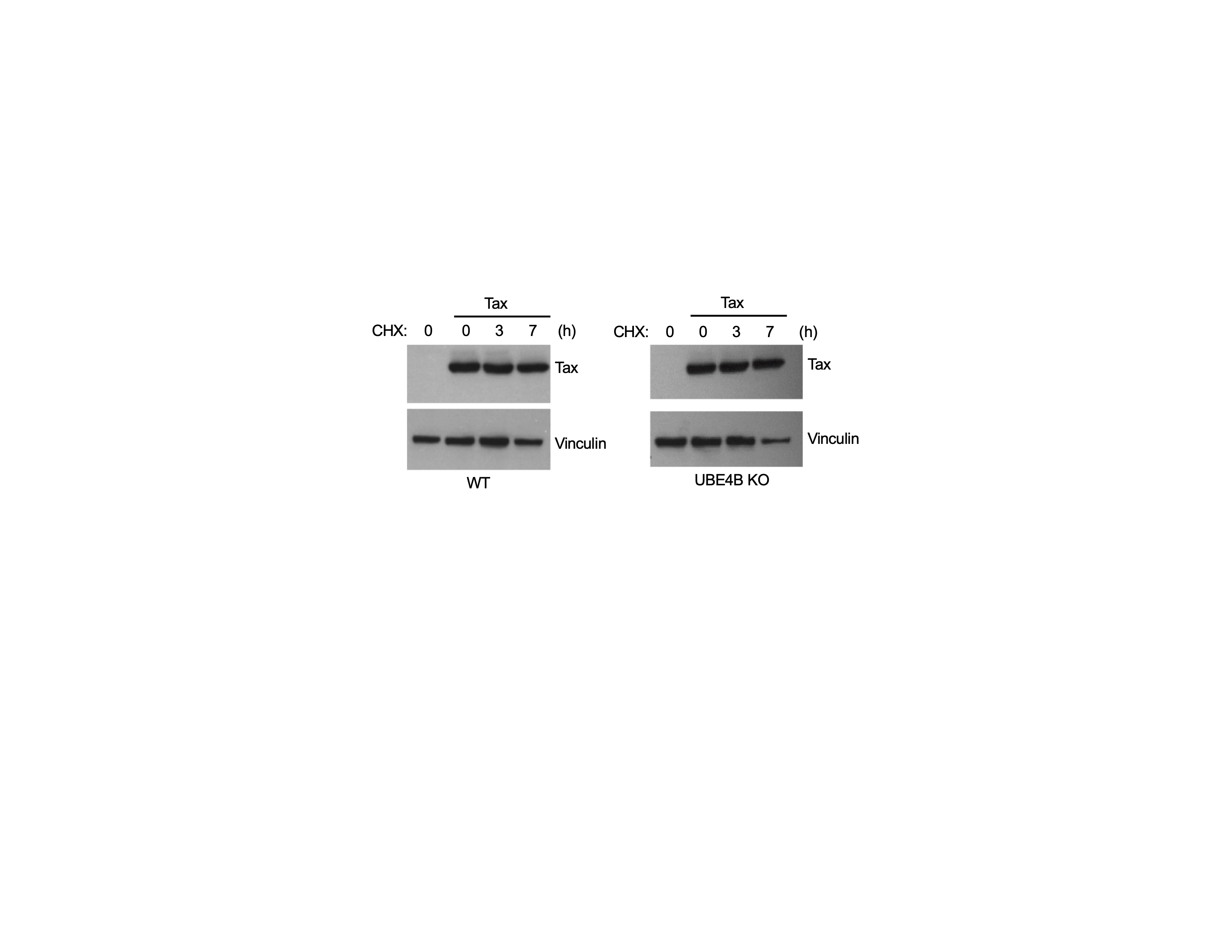

Supplement: S8 Fig — CHX chase assay with lysates from wild-type and UBE4B KO 293T cells (clone H1) transfected with Tax and treated with cycloheximide for the indicated times. Immunoblotting was performed with the indicated antibodies. (TIF) [file ppat.1008504.s008.tif]
