# Supplementary material for: The E3/E4 ubiquitin conjugation factor UBE4B interacts with and ubiquitinates the HTLV-1 Tax oncoprotein to promote NF-κB activation
Source: PLoS Pathog. 2020 Dec 23;16(12):e1008504. doi: 10.1371/journal.ppat.1008504 (PMC7790423; doi:10.1371/journal.ppat.1008504)
Supplement: S1 Table — (PDF) [file ppat.1008504.s009.pdf]

S1 Table. Oligonucleotides used in the study

Name	Forward or reverse	Sequences (5' to 3')
Tax	Forward	ATACCCAGTCTACGTGTTTGGAG
	Reverse	CCGATAACGCGTCCATCGATG
CD25	Forward	ATGCAAGAGAGGTTTCCGCA
	Reverse	AGTGGCAGAGCTTGTGCATTG
cIAP2	Forward	TTTCCGTGGCTCTTATTCAAACT
	Reverse	GCACAGTGGTAGGAACTTCTCAT
IRF4	Forward	GCACAGTGGTAGGAACTTCTCAT
	Reverse	AGGGTAAGGCGTTGTCATGG
UBE4B	Forward	GAGAAAAGCGGAGCCTCAGT
	Reverse	GGTCCTTCCAAGAGACACGG
18S	Forward	GTAACCCGTTGAACCCCATT
	Reverse	CCATCCAATCGGTAGTAGCG
UBE4B gRNA1	Forward	CACCGGGAGGAGCTGAGCGCTGATG
	Reverse	AAACCATCAGCGCTCAGCTCCTCCC
UBE4B gRNA2	Forward	CACCGAAGCCCCATGTTCTGCAGCG
	Reverse	AAACCGCTGCAGAACATGGGGCTTC
UBE4B gRNA3	Forward	CACCGGAACAAAGTTTTACCATGAG
	Reverse	AAACCTCATGGTAAAACTTTGTTCC
UBE4B Surveyor 1	Forward	ACCTGGGAAGCAGAGGGTAA
	Reverse	CTCCGTTCCTTCTCCAAGCC
UBE4B Surveyor 2	Forward	GGGGTCAGCTGGCTTATTCA
	Reverse	GCCATACCCTGTATGACACTGA
UBE4B Surveyor 3	Forward	ATTACTGGGGGTCCTGTCAGA
	Reverse	GCATGGCCAACACAGTGATTT
